# Pulmonary cancers across different histotypes share hybrid tuft cell/ionocyte-like molecular features and potentially druggable vulnerabilities

**DOI:** 10.1038/s41419-022-05428-x

**Published:** 2022-11-19

**Authors:** Yosuke Yamada, Djeda Belharazem-Vitacolonnna, Hanibal Bohnenberger, Christel Weiß, Naoko Matsui, Mark Kriegsmann, Katharina Kriegsmann, Peter Sinn, Katja Simon-Keller, Gerhard Hamilton, Thomas Graeter, Gerhard Preissler, German Ott, Sebastian Schölch, Naoki Nakajima, Akihiko Yoshizawa, Hironori Haga, Hiroshi Date, Roman K. Thomas, Iacopo Petrini, Giuseppe Giaccone, Philipp Ströbel, Alexander Marx

**Affiliations:** 1 Institute of Pathology, University Medical Centre Mannheim and Medical Faculty Mannheim, Heidelberg University, Mannheim, Germany; 2 Diagnostic Pathology, Kyoto University Hospital, Kyoto, Japan; 3 Institute of Pathology, University Medical Center Göttingen, University of Göttingen, Göttingen, Germany; 4 Department of Medical Statistics and Biomathematics, Medical Faculty Mannheim, Heidelberg University, Mannheim, Germany; 5 Institute of Pathology, University Hospital Heidelberg, Heidelberg, Germany; 6 Department of Hematology, Oncology and Rheumatology, University Hospital Heidelberg, Heidelberg, Germany; 7 Institute for Pharmacology, Medical University of Vienna, Vienna, Austria; 8 Thoracic Surgery, Klinik Löwenstein, Löwenstein, Germany; 9 Department of Thoracic Surgery, Klinik Schillerhöhe GmbH am Robert-Bosch-Krankenhaus, Stuttgart, Germany; 10 Department of Clinical Pathology, Robert-Bosch-Krankenhaus, and Dr. Margarete Fischer-Bosch Institute of Clinical Pharmacology, Stuttgart, Germany; 11 Department of Surgery, University Medical Centre Mannheim, University of Heidelberg, Mannheim, Germany; 12 Junior Clinical Cooperation Unit Translational Surgical Oncology (A430), German Cancer Research Center (DKFZ), Heidelberg, Germany; 13 DKFZ Hector Cancer Institute at University Medical Center Mannheim, Mannheim, Germany; 14 Department of Thoracic Surgery, Kyoto University Graduate School of Medicine, Kyoto, Japan; 15 Department of Translational Genomics, Medical Faculty, University of Cologne, Cologne, Germany; 16 Institute of Pathology, University of Cologne, Cologne, Germany; 17 German Cancer Consortium (DKTK), partner site Heidelberg and German Cancer Research Center (DKFZ), Heidelberg, Germany; 18 Department of Translational Research and New Technologies in Medicine, University Hospital of Pisa, Pisa, Italy; 19 Weill Cornell Medicine, Cornell University, New York, NY USA

**Keywords:** Lung cancer, Lung cancer, Oncogenes

## Abstract

Tuft cells are chemosensory epithelial cells in the respiratory tract and several other organs. Recent studies revealed tuft cell-like gene expression signatures in some pulmonary adenocarcinomas, squamous cell carcinomas (SQCC), small cell carcinomas (SCLC), and large cell neuroendocrine carcinomas (LCNEC). Identification of their similarities could inform shared druggable vulnerabilities. Clinicopathological features of tuft cell-like (tcl) subsets in various lung cancer histotypes were studied in two independent tumor cohorts using immunohistochemistry (*n* = 674 and 70). Findings were confirmed, and additional characteristics were explored using public datasets (RNA seq and immunohistochemical data) (*n* = 555). Drug susceptibilities of tuft cell-like SCLC cell lines were also investigated. By immunohistochemistry, 10–20% of SCLC and LCNEC, and approximately 2% of SQCC expressed POU2F3, the master regulator of tuft cells. These tuft cell-like tumors exhibited “lineage ambiguity” as they co-expressed NCAM1, a marker for neuroendocrine differentiation, and KRT5, a marker for squamous differentiation. In addition, tuft cell-like tumors co-expressed BCL2 and KIT, and tuft cell-like SCLC and LCNEC, but not SQCC, also highly expressed MYC. Data from public datasets confirmed these features and revealed that tuft cell-like SCLC and LCNEC co-clustered on hierarchical clustering. Furthermore, only tuft cell-like subsets among pulmonary cancers significantly expressed *FOXI1*, the master regulator of ionocytes, suggesting their bidirectional but immature differentiation status. Clinically, tuft cell-like SCLC and LCNEC had a similar prognosis. Experimentally, tuft cell-like SCLC cell lines were susceptible to PARP and BCL2 co-inhibition, indicating synergistic effects. Taken together, pulmonary tuft cell-like cancers maintain histotype-related clinicopathologic characteristics despite overlapping unique molecular features. From a therapeutic perspective, identification of tuft cell-like LCNECs might be crucial given their close kinship with tuft cell-like SCLC.

## Introduction

Tuft cells are epithelial cells with distinct microvilli (tufts) on the apical side. They occur in multiple organs and regulate immune functions, e.g., anti-parasitic immunity [1–4], and thymic T-cell development [5, 6]. In the intestine, they are sensors of chemical signals, including those from parasites. Through the secretion of mediators, including interleukin-25 and acetylcholine, they initiate anti-parasitic immune responses and regulate respiration [1–4]. Thymic tufts cells produce similar mediators and influence the thymic microenvironment, especially innate immunity [5–7].

Tuft cells have attracted attention in oncology after the discovery of a tuft cell-like small cell lung cancer (SCLC) subset, which exhibits a tuft cell-like gene expression signature [8], including *POU2F3*, the tuft cell master regulator [9]. Meanwhile, four molecular SCLC subtypes were delineated [10], and their features have been intensively investigated for personalized treatment options [11–16]. We identified tuft cell-like subsets also in non-small cell lung cancers (NSCLCs), including adenocarcinoma, squamous cell carcinoma (SQCC), and large cell neuroendocrine carcinoma (LCNEC), and in thymic carcinomas [17, 18].

Ionocytes are rare epithelial cells recently discovered in the lung [19, 20]. They maintain the fluid and mucus physiology of the airways and are a major source of CFTR activity (mutations of *CFTR* are the most common cause of cystic fibrosis [21]). *FOXI1* is the master regulator of pulmonary ionocytes [19, 20]. To our knowledge, ionocytes have not yet been discussed in relation to lung cancer.

Here, we elucidated clinicopathological and molecular features of pulmonary tuft cell-like cancers classified as SCLC, LCNEC, SQCC, and adenocarcinoma and found them to share an immature hybrid tuft cell/ionocyte-like and anti-apoptotic signature. Rare tuft cell-like SCLC cell lines showed susceptibility to inhibitors of BCL2 and PARP.

## Materials and methods

### Patient cohorts and immunohistochemistry

We examined two cohorts: (1) one from Japan, Kyoto University Hospital (cohort-J): 369 adenocarcinomas, 225 SQCCs, 36 SCLC, and 44 LCNECs, and (2) one from Germany, University Medical Center Göttingen (cohort-G): 47 SCLCs and 23 LCNECs. When SCLC and LCNEC are addressed together, they will be collectively labeled as neuroendocrine carcinoma (NEC).

We performed immunohistochemistry (IHC) on formalin-fixed, paraffin-embedded specimens of whole sections or tissue microarrays with the Benchmark Ultra immunostainer (Ventana Medical Systems, Tucson, AZ, USA). The primary antibodies used are described in Fig. S1A. The positive ratio (%) of tumor cells was estimated for each antibody. Considering the histogram of immunoreactive tumor cells in SQCC (Fig. S1B), the IHC for POU2F3 was interpreted as positive when ≥10% of the tumor cells exhibited nuclear staining.

### Publicly available datasets and identification of tuft cell-like subsets

We utilized four centrally reviewed datasets of lung cancers; (1) 230 adenocarcinomas (TCGA, Nature 2014), (2) 178 SQCCs (TCGA, Nature 2012), (3) 81 SCLCs (U Cologne, Nature 2015), and (4) a dataset of 66 LCNECs [27] (datasets 1–3 are archived in cBioPortal [cbioportal.org]) [22–27]. As described previously [17], we extracted tuft cell-like SQCC and adenocarcinoma cases from the above datasets with mRNA expression Z scores of >2 of both *POU2F3* and *GFI1B*, while tuft cell-like SCLC and LCNEC were extracted by the histogram of *POU2F3* and *GFI1B* expressions [17], and the subsequent confirmation of strong expression of other tuft cell-markers [8].

### Cell culture and MTT assay

Six SCLC cell lines, i.e., NCI-H69, NCI-H211, NCI-H526, NCI-H1048, UHGc5, and SCLC26A, were used in the study. MTT assays were performed with cells that were plated in 96-well plates at 1 × 10^4^ cells/well with 100 μl of appropriate media containing variable concentrations of Olaparib (Axon Medchem, Groningen, The Netherlands), Talazoparib (Selleck, Houston, TX, USA), Venetoclax (Selleck), and Navitoclax (Selleck). The details and evaluation of IC50 of drugs and the combined effects of two drugs are described in the supplement.

### Western Blotting and real-time quantitative PCR

Details of Western blotting and real-time quantitative PCR are given in the supplement.

### Statistical analyses

Statistical analyses that were performed in the study are described in the supplement.

### Ethical approval

The study was approved by the local Ethics Committee II, University of Heidelberg (2018-516N-MA), and the Medical Ethics Committees of the Kyoto University Graduate School of Medicine and Kyoto University Hospital (R3081).

## Results

### Clinicopathological features of tuft cell-like lung cancers in two independent cohorts

Clinicopathological features of tuft cell-like lung cancers were investigated in a Japanese cohort (cohort-J) and a German cohort (cohort-G) and are reported separately to account for differences between them (Fig. S2A, B).

**Fig. 1:**
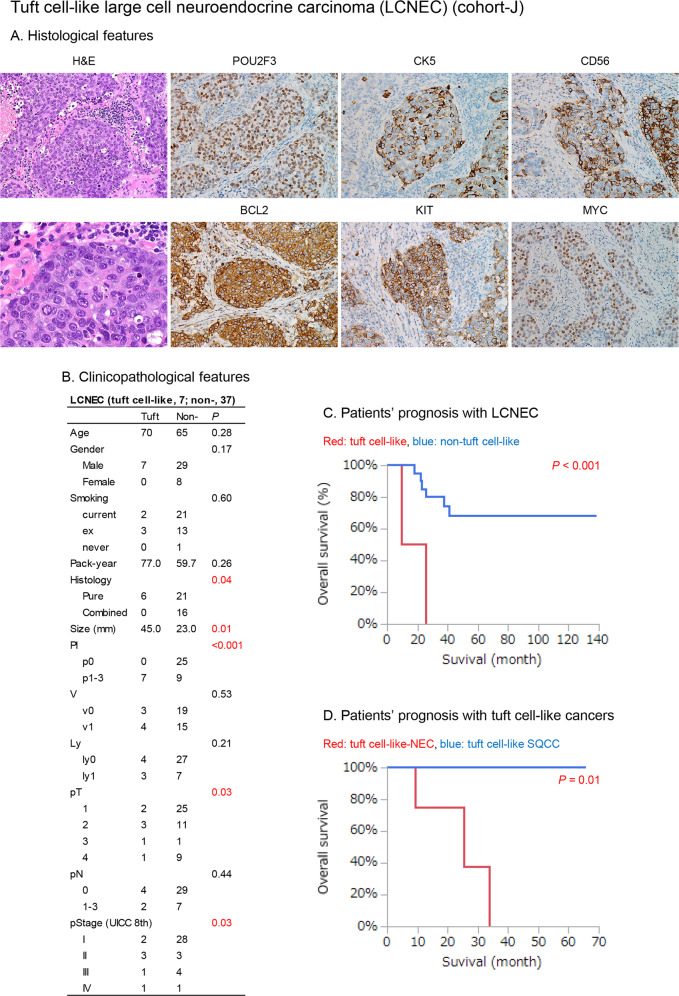
Clinicopathological findings of tuft cell-like large cell neuroendocrine carcinoma (LCNEC) and patients’ prognosis with tuft cell-like cancers (cohort-J). **A** The histology of a tuft cell-like LCNEC (case no.12 in Fig. S2). The tumor shows a nested growth pattern and vague rosetting. The tumor cells show non-small cell lung cancer cytology (conspicuous nucleoli, moderate amount of cytoplasm) and are diffusely positive for POU2F3, and focally positive for CK5 and CD56. The tumor cells are also positive for BCL2, KIT, and MYC. **B** Clinicopathological features. Pl, pleural invasion; V, vascular invasion (v0: −, v1: +); Ly, lymphatic invasion (ly0: −, ly1: +) **C**, **D** Patients’ prognosis. Tuft cell-like LCNECs exhibit a significantly poorer prognosis than non-tuft cell-like LCNECs (C). Tuft cell-like NECs (i.e., the joint tuft cell-like SCLCs and LCNECs, *N* = 12) exhibit a significantly worse prognosis than tuft cell-like SQCCs (*N* = 5) (D).

In cohort-J, 18 tuft cell-like lung cancers by POU2F3-IHC were identified (i.e., ≥10% of the tumor cells exhibited nuclear staining in 18 cases): 0/369 in adenocarcinoma, 5/220 (2.3%) in SQCC, 6/36 (16.7%) in SCLC, and 7/44 (15.9%) in LCNEC (Fig. 1A and Fig. S3). Tuft cell-like LCNEC had a larger size and an inferior prognosis (both *P* < 0.05) compared with non-tuft cell-like LCNEC (Fig. 1B, C), while the respective subsets of SCLCs and SQCCs showed no prognostic differences (Figs. S4 and S5). Patients with tuft cell-like NECs had a worse prognosis than patients with tuft cell-like SQCC *(P* < 0.05) (Fig. 1D).

Cohort-G (47 SCLCs and 23 LCNECs) contained 18 (38.3%) and 6 (26.1%) tuft cell-like SCLC and LCNECs, respectively. Tuft cell-like SCLC rather than tuft cell-like LCNEC were larger than their non-tuft cell-like counterparts (Fig. S6A), but there were no prognostic differences between tuft cell-like and non-tuft cell-like SCLC and LCNEC (Fig. S6B, C).

In both cohorts, tuft cell-like SCLC and tuft cell-like LCNEC showed no significant prognostic differences (Figs. S4D, S6D). However, multiple Cox regression analyses detected an interaction of tuft cell-like phenotype and histology (i.e., SCLC or LCNEC) with respect to patients’ prognoses in Cohort-J (Fig. S2C).

Overall, these results suggest that the tuft cell-like phenotype of pulmonary NECs may be associated with clinicopathologic features and prognosis; however, further studies are needed to support this hypothesis, as the number of cases was not large enough and the clinical characteristics of the two cohorts differed substantially. Conversely, histotype apparently remains important, as patients with tuft cell-like NECs and SQCCs showed different survival.

### Tuft cell-like lung cancers exhibit “lineage ambiguities”

**Fig. 2:**
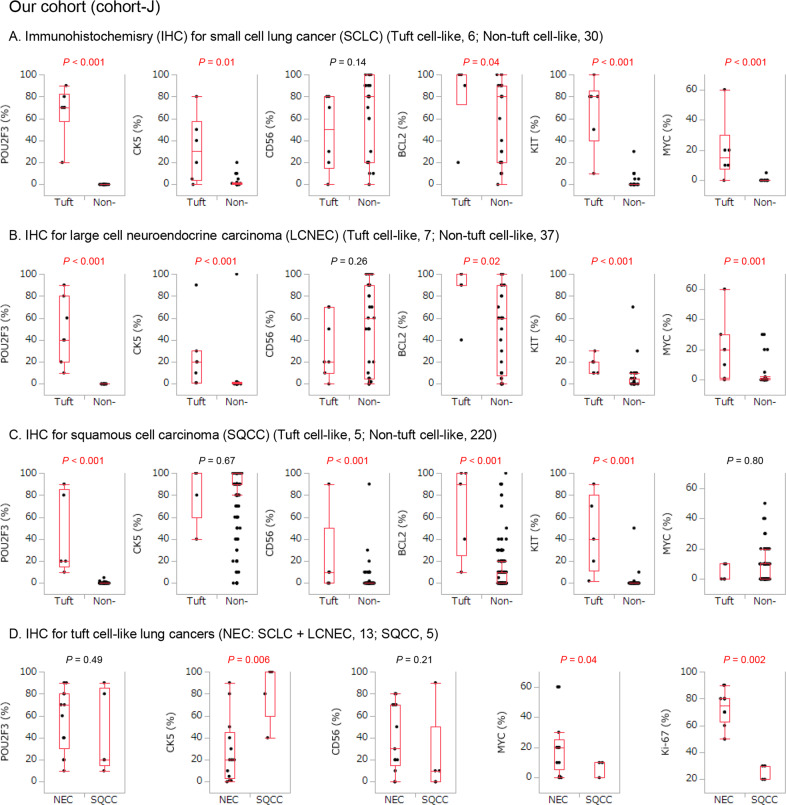
Tuft cell-like lung cancers and their immunohistochemical features (cohort-J). **A** Small cell lung cancer (SCLC); **B** large cell neuroendocrine carcinoma (LCNEC); **C** squamous cell carcinoma (SQCC); **D**, tuft cell-like neuroendocrine carcinomas (NECs) and SQCC. Compared to each non-tuft cell-like counterpart, tuft cell-like SCLCs and LCNECs show significantly higher percentages of cells expressing MYC, and the squamous differentiation marker, CK5. On the other hand, tuft cell-like SQCCs show significantly higher percentages of cells expressing CD56, a marker of neuroendocrine cells. All the tuft cell-like SCLCs, LCNECs, and SQCCs exhibit significantly higher positive ratios for BCL2 and KIT. The positive ratios for MYC and Ki-67 of tuft cell-like neuroendocrine carcinomas (NECs: SCLC + LCNEC) are significantly higher than those of SQCCs.

To understand the biased prevalence of tuft cell-like tumors among histotypes (SCLC ≥ LCNEC ≫ SQCC, and absence in adenocarcinoma), we examined the expression of a marker of squamous differentiation (CK5) and a common marker of neuroendocrine tumors (CD56) [28]. Interestingly, CK5 was diffusely expressed in almost all tuft cell-like SQCCs as expected but focally also in tuft cell-like NECs, and the difference in the expression between tuft cell-like and non-tuft cell-like NECs was significant (*P* < 0.01) (Fig. 2A, B). Conversely, CD56 was expressed in most tuft cell-like NECs, but also in a subset of tuft cell-like SQCCs, in which the percentages of CD56-positive cells were significantly higher than in non-tuft cell-like SQCC (*P* < 0.001) (Fig. 2C).

In contrast, most tuft cell-like lung cancers were negative for the highly specific neuroendocrine markers, chromogranin A and synaptophysin, and TTF1, a marker of pulmonary adenocarcinoma and SCLC (Fig. S3). These results resembled those reported for tuft cell-like SCLCs [29] and suggest “lineage ambiguities” of tuft cell-like lung carcinomas: while tuft cell-like NECs exhibited an attenuated neuroendocrine and a stronger squamous phenotype, tuft cell-like SQCC showed a stronger neuroendocrine phenotype than their non-tuft cell-like counterparts.

### Strong protein expression of BCL2, KIT, and MYC in pulmonary tuft cell-like cancers

We next analyzed the protein expression of oncogenes known to be transcriptionally upregulated in tuft cell-like lung cancer subsets, i.e., BCL2 [10, 16], MYC [15, 16, 30, 31], and KIT [17], because their expression might further delineate tuft cell-like variants and open new therapeutic perspectives.

Most of the pulmonary tuft cell-like cancers of Cohort-J strongly expressed BCL2 and KIT protein, and the percentages of immunoreactive cells were significantly higher in the tuft cell-like than non-tuft cell-like groups (*P* < 0.05) (Fig. 2A–C). Also, the percentages of MYC-positive tumor cells were significantly higher in tuft cell-like than non-tuft cell-like NECs (*P* < 0.001) (Fig. 2A, B), but not between tuft cell-like and non-tuft cell-like SQCCs (*P* = 0.80) (Fig. 2C). Among tuft-cell-like cancers, the percentage of MYC-positive cells and the Ki-67 labeling index were significantly higher in NECs than SQCCs (both *P* < 0.05) (Fig. 2D). In Cohort G, the findings resembled those obtained with the NECs of Cohort-J (Fig. S6E, F).

### Comparable prevalence and molecular features of tuft cell-like tumors in public datasets

We next analyzed publicly available datasets that were centrally reviewed regarding histological diagnosis, to evaluate the above findings. Similar to our previous study [17], strong co-expression of *POU2F3* and *GFI1B*, characteristic of non-neoplastic tuft cells, was the criterion to identify 1 (0.4%) tuft cell-like adenocarcinoma and 3 (1.7%) tuft cell-like SQCCs in the centrally reviewed “bona fide” cohorts of the TCGA adenocarcinoma and SQCC datasets [22, 23, 25, 26] (Fig. S7A). These percentages are comparable to those in our cohort-J. In the LCNEC [27] and SCLC [24] datasets, there were 12 tuft cell-like LCNECs (18.2%) (as reported previously [17]) and 11 tuft cell-like SCLS (13.6%) (Fig. S7B, C). All tuft cell-like cancers identified in this way also strongly expressed other tuft cell-markers, such as *TRPM5*, *SOX9*, *ASCL2*, and *AVIL* [8] but not *CHAT* (Fig. S7A–C).

**Fig. 3:**
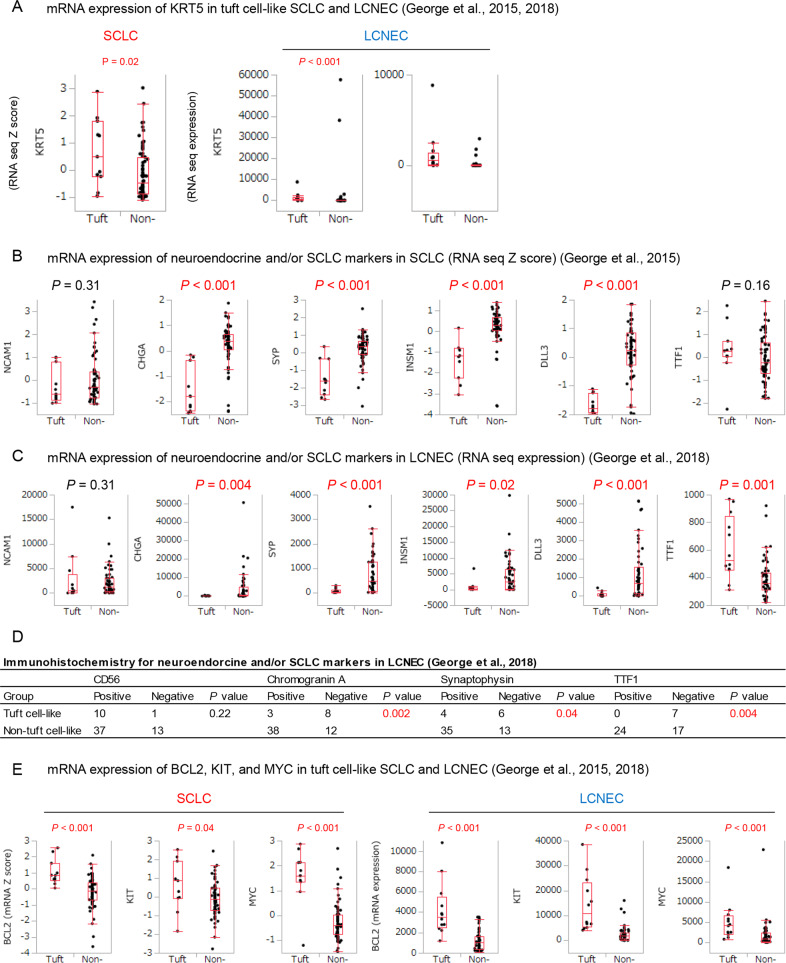
Expression profiles of tuft cell-like lung cancers. Transcriptional and immunohistochemical features of tuft cell-like lung cancers retrieved from public datasets (**A**, **B**, **E** [24]. **A**, **C**, **D**, **E** [27]) (FPKM, fragments per kilobase of exon per million reads mapped).

Consistent with our protein expression analyses, data from the public datasets [24, 27] confirmed “lineage ambiguities”: In both tuft cell-like SCLC and LCNEC, *KRT5* mRNA expression levels were significantly higher (Fig. 3A), while expression levels of specific neuroendocrine markers (*CHGA*, *SYP*, and *INSM1*), and *DLL3*, a Notch ligand with therapeutic relevance in SCLC [29], were significantly lower than in their non-tuft cell counterparts (Fig. 3B, C). Following the same line, tuft cell-like SQCC tended to show higher mRNA levels of *NCAM1* than non-tuft cell-like SQCC (Fig. S8A). Moreover, immunohistological features reported in the public LCNEC dataset [27] confirmed our findings: tuft cell-like LCNEC expressed chromogranin A, synaptophysin, and TTF1 less frequently than non-tuft cell-like LCNECs (*P* < 0.05), while almost all tuft cell-like LCNECs expressed CD56 (Fig. 3D). Paradoxically, although tuft cell-like LCNECs did not express TTF1 protein (Fig. 3D), their *TTF1* mRNA levels were remarkably high (Fig. 3C) for unknown reasons.

Finally, analysis of the public databases [24, 27] confirmed that *BCL2*, *KIT*, and *MYC*, were upregulated in both tuft cell-like SCLC and LCNEC (Fig. 3E), and that tuft cell-like compared to non-tuft cell-like SQCCs expressed higher levels of *BCL2* and *KIT* (*P* < 0.05), while expression levels of *MYC* were not statistically different (*P* = 0.09) (Fig. S8A). Importantly, the similar prognosis of tuft cell-like SCLC and LCNEC was confirmed as well (Fig. S8B).

### Tuft cell-like SCLC and LCNEC form a joint cluster on mRNA hierarchical clustering

**Fig. 4:**
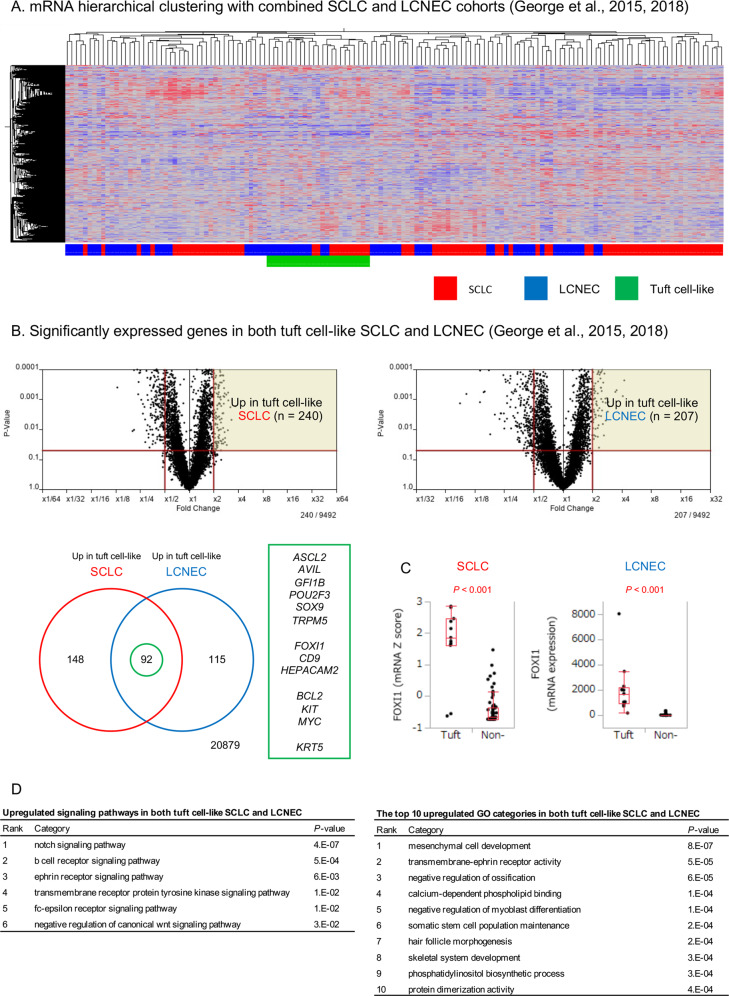
mRNA expression of tuft cell-like small cell lung cancer (SCLC), pulmonary large cell neuroendocrine carcinoma (LCNEC), and squamous cell carcinoma (SQCC) ([24, 27], TCGA Nature 2012). **A** Unsupervised mRNA expression clustering with the combined SCLC and LCNEC cohort. **B** Significantly expressed genes in both tuft cell-like SCLC and LCNEC, compared with the respective non-tuft cell-like counterparts (>2 folds, *P* < 0.05). *POU2F3*, *GFI1B*, *TRPM5*, *SOX9*, *ASCL2*, *AVIL*, representative tuft cell genes; *FOXI1*, the master regulator of ionocytes; *BCL2*, *KIT*, and *MYC*, well-known oncogenes; *KRT5*, a marker of squamous differentiation, were included among the 92 genes. **C** *FOXI1* mRNA expression in tuft cell-like SCLC and LCNEC. The numbers of the *y*-axis indicate mRNA expression Z score in SCLC and FPKM (fragments per kilobase of exon per million reads mapped) in LCNEC. **D** The pathways and gene ontology (GO) analyses for upregulated genes in tuft cell-like SCLC and LCNEC. Genes related to the Notch signaling pathway were most significantly enriched.

To further understand the similarities between tuft cell-like SCLC and LCNEC, the combined SCLC and LCNEC mRNA datasets [24–27] were subjected to unsupervised clustering. This revealed that SCLCs and LCNECs are separated into several clusters and that one cluster contains all the tuft cell-like SCLCs/LCNECs without inclusion of any non-tuft cell-like tumors. However, these tuft cell-like SCLCs and LCNECs were not randomly distributed within the cluster but formed two subclusters largely according to their small cell versus large cell histotype (Fig. 4A).

To address the resulting hypothesis that tuft cell-like SCLC and LCNEC are more closely related to each other than to their non-tuft cell-like histological counterparts, we extracted upregulated genes in both tuft cell-like SCLC and LCNEC compared with the respective non-tuft cell-like counterparts (>2 folds, *P* < 0.05) and performed GO (gene ontology) analysis.

Consistent with the criteria of tuft cell-like tumors and our above findings, representative tuft cell genes (e.g., *POU2F3*, *GFI1B*, and *TRPM5*) [8], as well as *KRT5*, *BCL2*, *KIT*, and *MYC* were among the 92 genes that were upregulated both in tuft cell-like SCLC and tuft cell-like LCNEC (Fig. 4B-C). As to pathways differentially enriched both in tuft cell-like SCLC and LCNECs, the Notch signaling pathway was top-ranked among the activated pathways (Fig. 4D). This fits the classification of all tuft cell-like LCNECs as type II LCNECs [17], which typically exhibit active Notch signaling [27]. Moreover, the top-ranked activated pathways in pulmonary tuft cell-like NECs reflect the conditions required for tuft cell development from pulmonary basal cells (active NOTCH and inactive WNT signaling) [32], and hint to potential vulnerability towards inhibitors of NOTCH pathway constituents [33], Bruton’s tyrosine kinase [12], Ephrin receptor signaling [34], and receptor tyrosine kinases [16].

Subsequently, we focused on genes with differential expression between tuft cell-like SCLC and tuft cell-like LCNEC (*n* = 96 and 142, respectively [>2 folds, *P* < 0.05]). On the GO analyses (Fig. S8C), genes related to inflammation and cytokines were enriched in tuft cell-like SCLC, while genes related to neural differentiation/development were enriched in tuft cell-like LCNEC (Fig. S8D). Thus, tuft cell-like NECs form a distinct tumor group among pulmonary NECs, but tuft cell-like SCLC and tuft cell-like LCNEC remain distinguishable tumor types.

### Tuft cell-like lung cancers exhibit a hybrid tuft cell/ionocyte-like signature

Unexpectedly, we found that *FOXI1*, the ionocyte master regulator [19, 20], and *CD9*, an ionocyte-specific gene [20], were among the 92 significantly upregulated genes in tuft cell-like NECs compared to non-tuft cell-like NECs (Fig. 4B, C and S8E). *HEPACAM2*, a marker of renal intercalated cells [35], which functionally resemble ionocytes, was also included (Fig. S8E). On the other hand, *CFTR*, the most representative marker of mature ionocytes [20] was not contained (Fig. S8E). Significant expression of *FOXI1* and *HEPACAM2* was also observed in tuft cell-like SQCCs in the TCGA dataset (Fig. S8E).

### Tuft cell-like SCLC cell lines are sensitive to PARP and BCL2 inhibitors

**Fig. 5:**
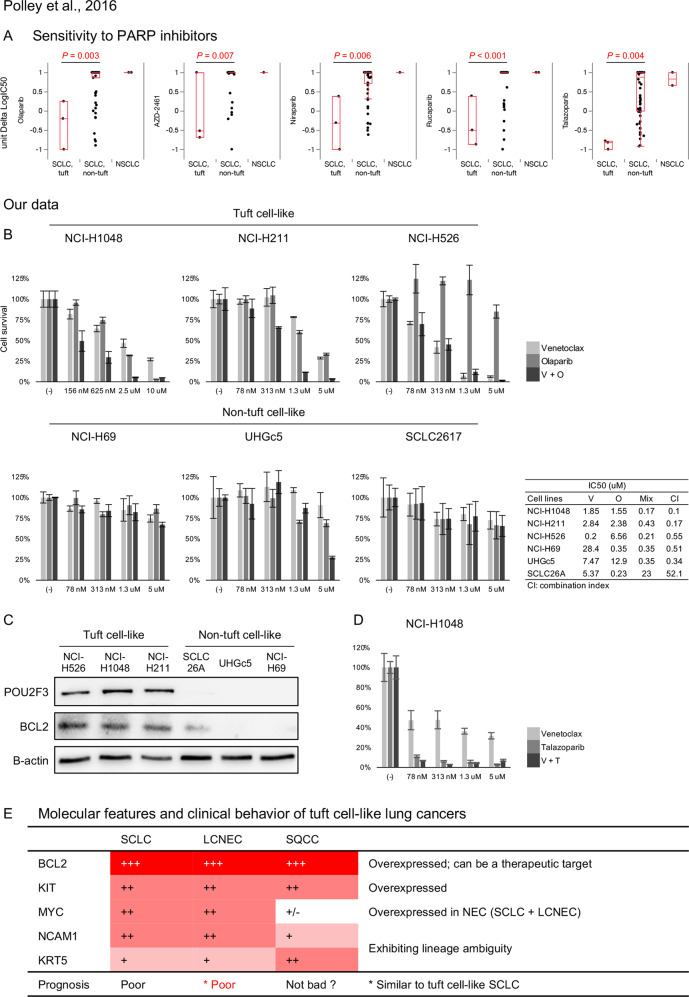
PARP and BCL2 inhibitors preferentially affect tuft cell-like compared to non-tuft cell-like small cell lung cancers (SCLC) in vitro (A: Polley et al., 2016. B-D: our data). **A** Sensitivity of SCLC cell lines to PARP inhibitors (PARPi) [35] (Tuft cell-like SCLCs, 3 cell lines; Non-tuft cell-like SCLCs, 64; Non-SCLCs, 3). The vertical line means unit Delta LogIC50 (−1 to 1); the smaller the number, the better the response of the cell line. Tuft cell-like SCLC cell lines showed significantly better response to all five different PAPRi (AZD-2461, Niraparib, Olaparib, Rucaparib, and Talazoparib) than non-tuft cell-like SCLC cell lines. **B** MTT assay-based survival analysis of our limited set of SCLC cell lines (*n* = 6). Tuft cell-like SCLC cell lines (*n* = 3) showed a better response to the BCL2-inhibitor (BCL2i), Venetoclax (V), the PARPi, Olaparib (O) (except for NCI-H526 cells), and their combination (Mix) than the non-tuft cell-like cell lines (*n* = 3). Combination indexes (CIs) <1 by the Chou-Talalay Method indicate synergistic effects of the combination therapy in 5 of the 6 cell lines, especially for NCI-1048 and NCI-H211. **C** Western blotting for POU2F3 and BCL2 in the six SCLC cell lines (with Beta-actin as housekeeping). Tuft cell-like SCLC cell lines clearly express POU2F3 and BCL2 at the protein level, while non-tuft cell-like SCLC cell lines do not express POU2F3 or BCL2, except for a weak expression of BCL2 in SCLC26A. **D** MTT assay-based survival analysis of the tuft cell-like SCLC cell line, NCI-H1048, showed a striking response to the PARP-inhibitor, Talazoparib (compare with the much poorer response to Olaparib in Fig. 5B). **E** Summary of molecular features and clinical behavior of tuft cell-like lung cancers. The scores (+/−, +, ++, +++) were estimated based on mRNA and protein expression levels.

Last, we asked whether different tuft cell-like lung cancers might share the same vulnerabilities towards anti-cancer drugs in vitro. We had to restrict this study to SCLC because, among all published cell lines, there are only four with a tuft cell-like phenotype in the NCI collection, and all are derivatives of SCLCs [8]. Having spotted three of them among 67 SCLCs with known sensitivities towards a broad drug library (the fourth cell line, COR-L311, was not included in the study) [36], we identified PARP inhibitors as the only class of inhibitors to which the three tuft cell-like SCLC cell lines (NCI-H211, NCI-H526, NCI-H1048) were significantly more sensitive than the non-tuft cell-like SCLC lines (Fig. 5A). This conclusion is consistent with a recent report by Gay et al. [12]. However, when validating this in silico finding in an in vitro experiment, we observed only moderate effects at therapeutically relevant [37] Olaparib concentrations (Fig. 5B).

Thus, we speculated whether combining with another inhibitor might improve the killing effect. Among the two candidates with high expression in tuft cell-like lung cancers, i.e., BCL2 (Fig. 5C and S9a) and KIT, we selected BCL2 because (1) BCL2 inhibitors (e.g., Venetoclax [ABT-199]) are in clinical use; (2) KIT inhibition is ineffective in KIT-wildtype tumors [38], while such a relationship has not been established in BCL2; (3) BCL2 inhibition has been proposed for SCLC [39], especially ASCL1-SCLC [12], suggesting that it may be clinically relevant in tuft cell-like SCLC as well. When combining Olaparib with Venetoclax, we observed synergistic effects (i.e., CIs < 1) in five of the six SCLC cell lines by the Chou Talalay method, but the effects were most obvious in two of the three tuft cell-like SCLC cell lines (CI = 0.10 in NCI-H1048, and 0.17 in NCI-H211) (Fig. 5B).

Treatment with another approved PARP-inhibitor, Talazoparib that is presumably more potent than Olaparib due to alternative targets [40–42], showed a strikingly higher sensitivity to Talazoparib in tuft cell-like NCI-H1048 cells (for unknown reasons) and, to a much lesser extent, in non-tuft cell-like UHGc5 cells (Figs. 5D and S9B). A broadly effective, pre-clinical BCL2 family inhibitor, Navitoclax, did not change this tendency (Fig. S9C).

## Discussion

The new findings here are (i) pulmonary tuft cell-like cancers across various histotypes have considerably overlapping gene expression profiles, including a hybrid tuft cell/ionocyte-like signature; (ii) tuft cell-like NECs and SQCC nevertheless exhibit distinct histotype-associated clinicopathological features; (iii) in vitro, tuft cell-like SCLCs show higher vulnerability to PARP/BCL2 co-inhibition than to either drug alone.

The close kinship among tuft cell-like lung cancers was highlighted by the clustering analysis with combined SCLC and LCNEC datasets, because tuft cell-like SCLC and LCNEC formed a single, small cluster. Also, all the tuft cell-like lung cancer subtypes significantly expressed BCL2 and KIT, and tuft cell-like NECs unlike non-tuft cell-like NECs overexpressed MYC. In addition, tuft cell-like SCLC, LCNEC, and SQCC exhibited “lineage ambiguity”, namely with expressions of NCAM1 (NECs > SQCC) and KRT5 (SQCC > NECs) and infrequent expression of most neuroendocrine markers (Fig. 5D). Another facet of “lineage ambiguity” was the strong expression of FOXI1 in tuft cell-like cancers of different histotypes because this is the master regulator of ionocytes, which regulate airway surface physiology by expressing characteristic functional molecules, such as CFTR in the lung [19, 20]. Interestingly, co-expression of POU2F3 and FOXI1 occurs in an immature common precursor of mature tuft cells and ionocytes in the respiratory tract [19], and transient Krt5 expression is a feature of maturing murine ionocytes arising from basal cells [20]. Therefore, “lineage ambiguity” may actually point to a maturation blockade of lung cancer cells with a hybrid tuft cell/ionocyte-like molecular signature compared to mature tuft cells and ionocytes. Three other observations fit this hypothesis: (i) absence of tuft cell morphology, namely of brush-type villi on lung cancer cells [43, 44], (ii) poor expression of rare genes expressed by mature tuft cells (e.g., CHAT [choline acetyltransferase] (Fig. S7), and DCLK1 [not shown]) [45], and (iii) poor expression of genes of mature ionocytes (e.g., CFTR) [20].

On the other hand, we also noticed differences between tuft cell-like NECs and SQCCs, e.g., overexpression of MYC in tuft cell-like NECs but not in tuft cell-like SQCC. Between the two groups, Ki-67 immunohistochemistry was also significantly different. These findings may be related to the poorer prognosis of tuft cell-like NECs compared with SQCCs and underscore that pathological classification remains essential for patient management. Regarding tuft cell-like SCLC and LCNEC, inflammatory signals were enriched in the former and neural differentiation programs in the latter. A more differentiated nature of LCNEC seems consistent with its morphological features, such as the more abundant cytoplasm than SCLC. The inflammatory nature of tuft cell-like SCLC may warrant further studies under (immuno-)therapeutic perspectives [16].

Last, we proposed a therapeutic option for tuft cell-like lung cancers, i.e., co-inhibition of PARP and BCL2. The efficacy of PARP inhibitors for tuft cell-like SCLC was also recently proposed and discussed (e.g., concerning SLFN11, a biomarker of PARP inhibition [36, 46–50]) [12], but is not fully understood. Considering our in vitro findings, PARPi alone may not be sufficient to kill tuft cell-like SCLCs but be effective as part of a combination therapy [51]. Our findings suggest that tuft cell-like SCLCs with strong BCL2 expression might be particularly suitable for PARP/BCL2 co-inhibition, which may provide a rationale for applying this strategy to NSCLCs, especially LCNEC.

In parallel, functional studies with tuft cell-like SCLC cell lines, and comprehensive genetic and epigenetic profiling of tuft cell-like lung cancers are necessary to provide mechanistic evidence for the unique drug sensitivity of tuft cell-like SCLC; publicly available databases, e.g., SCLC-CellMiner [16] will be of help for such analyses. The hypersensitivity of H1048 cells to Talazoparib compared to Olaparib, which was unrelated to abnormal PARP16 levels [40] (Fig. S10), should also be investigated, because it remains unclear whether this hypersensitivity is attributable to the tuft cell-like phenotype. Finally, HPF1, a novel PARP1/2-interacting protein [41] and strong modifier of PARPi sensitivity [52], warrants study in tuft cell-like NECs, although on average, they did not show abnormal HPF1 expression levels or *HPF1* mutations in public datasets [24, 27] (not shown).

The current study has limitations. The restriction to resection specimens implies a selection bias, as most lung cancers are inoperable but biopsied before neoadjuvant approaches [53]. This bias may contribute to the clinicopathological differences between cohorts-J and -G (Fig. S2), although ethnic differences cannot be excluded. To resolve these issues, prospective clinical studies should include core needle biopsies from the full spectrum of lung cancers for molecular testing. Furthermore, only six SCLC cell lines were used in the drug experiments, which may be insufficient to infer the unique drug sensitivity of tuft cell-like lung cancers. Given the paucity of tuft cell-like cancer cell lines [12, 16, 36], in vivo cell line-based and patient-derived xenograft experiments are needed to validate the particular in vitro drug sensitivity of tuft cell-like SCLC.

Accumulating evidence suggests that tuft cell-like SCLCs are biologically distinct from the other SCLC subtypes [11, 16, 29, 54, 55]. Our study further underlines the uniqueness of this variant. Although further pre-clinical studies should be conducted, strong similarities of tuft cell-like LCNEC to tuft cell-like SCLC may justify their eligibility for inclusion in future SCLC clinical studies, particularly trials including PARP inhibitors or co-inhibition of PARP and BCL2.

## Supplementary information

Supplementary information

Raw data of WB of Fig. 5 and S10

## Data Availability

The datasets generated during and/or analyzed during the current study are available from the corresponding author on reasonable request.
