# Supplementary material for: Pulmonary cancers across different histotypes share hybrid tuft cell/ionocyte-like molecular features and potentially druggable vulnerabilities
Source: Cell Death Dis. 2022 Nov 19;13(11):979. doi: 10.1038/s41419-022-05428-x (PMC9675833; doi:10.1038/s41419-022-05428-x)

Supplementary information for

Pulmonary cancers across different histotypes share hybrid tuft cell/ionocyte-like molecular features and potentially druggable vulnerabilities

Yosuke Yamada, Djeda Belharazem-Vitacolonnna, Hanibal Bohnenberger, Christel Weiß, Mark Kriegsmann, Katharina Kriegsmann, Peter Sinn, Katja Simon-Keller, Gerhard Hamilton, Thomas Graeter, Gerhard Preissler, German Ott, Sebastian Schölch, Naoki Nakajima, Akihiko Yoshizawa, Hironori Haga, Hiroshi Date, Roman K. Thomas, Iacopo Petrini, Giuseppe Giaccone, Philipp Ströbel, Alexander Marx

Corresponding author

Yosuke Yamada, MD, PhD

Department of Diagnostic Pathology, Kyoto University Hospital

54 Shogoin Kawahara-cho, Sakyo-ku, Kyoto 606-8507, Japan

yyamada@kuhp.kyoto-u.ac.jp

Supplementary methods

Cell culture

Six SCLC cell lines, i.e., NCI-H69, NCI-H211, NCI-H526, NCI-H1048, UHGc5, and SCLC26A, were used in the study. They were kindly provided by G. Hamilton, Vienna, Austria (NCI-H69, NCI-H526, UHGc5, and SCLC26A) and I. Dahmen, Cologne, Germany (NCI-H211 and NCI-H1048), and all the cell lines were recently authenticated. NCI-H1048 was cultured with HITES medium (DMEM:F-12 medium [ThermoFisher Scientific, Waltham, MA, USA], 1x Insulin-Transferrin-Selenium [ThermoFisher Scientific], 10 nM Hydrocortisone [ThermoFisher Scientific], 10 nM beta-estradiol [ThermoFisher Scientific], and 2mM L-Glutamine Solution [ThermoFisher Scientific]) with 10% FCS, 100 U/ml penicillin-streptomycin (ThermoFisher Scientific) in 5% CO2 at 37 degrees. The other cell lines were cultured in RPMI1640 (ThermoFisher Scientific) under otherwise identical conditions as NCI-H1048.

MTT assay

Cells were plated in 96-well plates at 1x10^4 cells/well with 100 ul of appropriate media containing variable concentrations of Olaparib (Axon Medchem, Groningen, The Netherlands), Talazoparib (Selleck, Houston, TX, USA), Venetoclax (Selleck), and/or Navitoclax (Selleck). Each concentration was evaluated in triplicates. After culturing for 96

hours, 10 µl of 5 mg/ml of MTT solution (Carl Roth, Karlsruhe, Germany) was added to each well. After incubation for 3 hours in 5% CO2 at 37 degrees, the supernatant was discarded or, if the cells were growing in suspension, i.e., NCI-H69, NCI-H211, NCI-H526, UHGc5, and SCLC26A, the supernatant was aspirated after centrifugation of the plates at 3000 rpm for 5 minutes. Then, 100 µl of DMSO was added to each well. After incubation at room temperature for 30 minutes and shaking for 90 seconds, absorbance at OD=560 nm (reference OD = 620 nm) was read with a Tecan M200 plate reader (Tecan, Männedorf, Switzerland).

We calculated IC50 of a drug (or drugs) in each experiment using an online tool, https://www.aatbio.com/tools/ic50-calculator, with two parameter mode. Then, we utilized the Chou-Talalay Method [1] to evaluate the combined effects of two drugs. Combination indexes (CIs) were interpreted as 1, additive; < 1, synergistic; > 1, antagonistic. All experiments were replicated twice. Error bars represent s.d..

Western Blotting

Protein extraction was performed using radioimmunoprecipitation assay buffer (Thermo Fisher Scientific). Proteins were separated on 10% sodium dodecyl (lauryl) sulfate-polyacrylamide gels and were transferred to a polyvinylidene fluoride membrane (GE Healthcare, Chicago, IL). The membranes were blocked in 5% low-fat milk for 30 minutes at room temperature. Antibodies were diluted 1-to-1000 in tris-buffered saline with Tween 20,

incubated overnight at 4C for the primary antibody to POU2F3, BCL2, and Beta-actin (C4, Santra Cruz Biotechnology, Dallas, TX, USA) for 1 hour at room temperature for the secondary antirabbit immunoglobulin G, horseradish peroxidase–linked antibody (Cell Signaling Technology, Danvers, MA). The proteins were visualized using Pierce ECL Plus Western Blotting Substrate (Thermo Fisher Scientific) and the Fusion SL Imaging system (Peqlab Biotechologie, Erlangen, Germany).

Full length western blots are uploaded as a single "Supplemental Material" file.

Real-time quantitative PCR

We examined mRNA expression levels using real-time quantitative PCR (RT-qPCR) in the six SCLC cell lines. Total RNA was extracted using TRIzol (Thermofisher Scientific) and was reverse-transcribed using the PrimeScript RT reagent KIT (TaKaRa, Kusatsu, Japan). The RT-qPCR was performed using SYBR Premis Ex Taq (TaKaRa) and analyzed using a Step-One Real-Time PCR System (Thermofisher Scientific). Beta-2 microglobulin was used as a housekeeping gene, and its level of expression in the tonsil was set as 1. Primer pairs that were used for amplification are shown in Figure S1C.

Statistical analyses

Differences in continuous variables were evaluated by the Student t-test when the

variance followed a standard normal distribution, and by Welch's t-test or the Wilcoxon test when the variance did not follow a standard normal distribution (two-sided). Differences in categorical variables were evaluated by the chi-square test (two-sided). Kaplan-Meier survival curves were compared using the log-rank test. Unsupervised expression clustering with the combined SCLC and LCNEC datasets and the subsequent gene ontology (GO) analyses were performed with Subio Platform (https://www.subioplatform.com/products/subioplatform). Furthermore, univariable and multiple Cox regression analysis was applied to evaluate survival times considering several variables simultaneously and test for interactions. In the case of interactions between two factors, separate analyses were performed for each category of the relevant factor [2]. Statistical results with $P < 0.05$ were considered to be significant. All statistical analyses were performed using JMP14 or SAS, release 9.4 (SAS, Cary, NC, USA).

Supplementary figure legends

Figure S1. Antibodies and primers used in the study

A) Antibodies used in the study

B) The histogram of immunohistochemistry for POU2F3 in squamous cell carcinoma (SQCC) (our cohort, cohort-J)

C) Primers for RT-PCR used in the study

Figure S2. Multiple regression models in terms of patient survival

A) Prognoses of patients with pulmonary neuroendocrine carcinoma in cohort-J and cohort-G. Patient survivals significantly differ between the two cohorts.

B) Factors influencing patient survival (univariate analysis). Tuft cell-like phenotype inversely influence patient survival between cohort-J and cohort-G.

C) Multiple regression models. In cohort-J, an interaction between tuft cell-like phenotype and histology was detected (P = 0.039). No interaction was detected in cohort-G. Ly, lymphatic invasion; V, vascular invasion

Figure S3. Tuft cell-like lung cancers and their immunohistochemical features (cohort-J)

Immunohistochemical expression profiles of individual pulmonary tuft cell-like small cell lung cancers (SCLC), large cell neuroendocrine carcinomas (LCNEC), and squamous

cell carcinomas (SQCC). Each number indicates the percentage of immunoreactive tumor cells.

Figure S4. Clinicopathological findings of tuft cell-like lung small cell lung cancer (SCLC) (cohort-J)

A) Typical histological findings in a tuft cell-like SCLC (No. 2 in Figure S2). The tumor cells are diffusely positive for POU2F3 and BCL2, extensively positive for KIT, and focally positive for CK5, CD56, and c-Myc.

B) Demographic and pathological findings. Pl, pleural invasion; V, vascular invassion (v0: -, v1: +); Ly, lymphatic invasion (ly0: -, ly1: +)

C-D) Patients' prognoses between tuft cell-like and non-tuft cell-like SCLCs (C), and between tuft cell-like SCLC and tuft cell-like LCNEC (D), are not significantly different.

Figure S5. Clinicopathological findings of tuft cell-like squamous cell carcinoma (SQCC) (cohort-J)

A) The histology of a tuft cell-like SQCC (No.14 in Figure S2). The tumor cells are diffusely positive for POU2F3, CK5, p40 (the most reliable SQCC markers), CD56, BCL2, and weakly or focally positive for KIT and MYC.

B) Demographic and pathological findings. Pl, pleural invasion; V, vascular invassion (v0: -, v1: +); Ly, lymphatic invasion (ly0: -, ly1: +)

C) Patients' prognoses between tuft cell-like and non-tuft cell-like SQCCs are not significantly different.

Figure S6. Clinicopathological findings and prognoses of patients with tuft cell-like lung cancers (cohort-G)

A) Demographic and pathological findings. SCLC, small cell lung cancer; LCNEC, large cell neuroendocrine carcinoma; Pl, pleural invasion; V, vascular invassion (v0: -, v1: +); Ly, lymphatic invasion (ly0: -, ly1: +)

B-D) As to patients' prognosis, there were no significant differences between tuft cell-like and non-tuft cell-like SCLC and LCNEC (B-C). Also, tuft cell-like SCLC and tuft cell-like LCNEC showed no prognostic differences (D).

E-F) Both tuft cell-like SCLC and LCNEC show significantly higher expression of BCL2 and KIT than non-tuft cell-like counterparts. Tuft cell-like SCLC and LCNEC also trend towards a higher expression of MYC.

Figure S7. Expression profiles of tuft cell-like lung cancer (A: TCGA, Nature 2014 and 2012. B, C: George et al., 2015 and 2018).

A) mRNA expression of tuft cell-like pulmonary adenocarcinoma and squamous cell carcinoma (SQCC). The numbers indicate mRNA expression Z scores (TCGA, Nature 2014 and 2012)

B) mRNA expression of tuft cell-like small cell lung cancer (SCLC). The numbers indicate mRNA expression Z scores (George et al., 2015)

C) mRNA expression of tuft cell-like large cell neuroendocrine carcinoma (LCNEC) (George et al., 2018). The numbers indicate FPKM (fragments per kilobase of exon per million reads mapped). This data was cited from Yamada et al., 2021 [3].

Figure S8. Tuft cell-like small cell lung cancer (SCLC), large cell neuroendocrine carcinoma (LCNEC), and squamous cell carcinoma (SQCC) (A, C, D-F: George et al., 2018. B: TCGA Nature 2012. C-F: George et al., 2015)

A) mRNA expression in SQCC. The y-axis indicates mRNA expression Z score.

B) Patients' prognosis with tuft cell-like SCLC and LCNEC

C-D) Differentially expressed genes between tuft cell-like SCLC and LCNEC (C), and the respective enriched categories in GO analysis (D).

E) mRNA expression of ionocyte-related genes in tuft cell-like SCLC, LCNEC, and SQCC. The y-axis indicates mRNA expression Z scores in SCLC and SQCC, while FPKM (fragments per kilobase of exon per million reads mapped) in LCNEC.

Figure S9. qPCR and MTT assay-based analyses of six SCLC cell lines

A) qPCR-based analysis of our limited set of SCLC cell lines in terms of relative POU2F3 and BCL2 expression (with *Beta-2 microglobulin (B2M)* as housekeeping gene). Expression of tonsil was set to 1. Tuft cell-like SCLC cell lines highly express *POU2F3* and *BCL2*.

B-C) MTT assay-based survival analysis. A Tuft cell-like cell line, NCI-1048, shows strong sensitivity for Talazoparib. A non-tuft cell-like cell line, UHGc5, also shows relatively good response to Talazoparib.

Figure S10. qPCR and Western blotting for PARP1 and PARP16 in six SCLC cell lines

A) qPCR-based analysis of our limited set of SCLC cell lines in terms of relative PARP1 and PARP16 expression (with *Beta-2 microglobulin (B2M)* as housekeeping gene). PC3, a prostate cancer cell line, was used as a positive control.

B) Western blotting for PARP1 and PRPA16 in the SCLC cell lines (with Beta-actin as internal control). PC3 was used as a positive control.

In sum, PARP1 and PARP16 expression levels were not significantly different in tuft cell-like and non-tuft cell-like levels SCLC cell lines. PARP16 expression was not observed in protein levels, although PARP16 overexpression was recently identified as a molecular

mechanism (target) underlying the much higher sensitivity of PARP16-high tumor cells to Talazoparib compared to Olaparib [4].

References

1. Chou TC, Talalay P. Quantitative analysis of dose-effect relationships: the combined effects of multiple drugs or enzyme inhibitors. *Adv Enzyme Regul* 1984, **22:** 27-55.

2. Allison PD. *Survival Analysis Using SAS: A Practical Guide.*, Second edn. SAS Press: Cary, North Carolina, 2010.

3. Yamada Y, Simon-Keller K, Belharazem-Vitacolonnna D, Bohnenberger H, Kriegsmann M, Kriegsmann K*, et al.* A tuft cell-like signature is highly prevalent in thymic squamous cell carcinoma and delineates new molecular subsets among the major lung cancer histotypes. *J Thorac Oncol* 2021, **16**(6): 1003-1016.

4. Palve V, Knezevic CE, Bejan DS, Luo Y, Li X, Novakova S*, et al.* The non-canonical target PARP16 contributes to polypharmacology of the PARP inhibitor talazoparib and its synergy with WEE1 inhibitors. *Cell Chem Biol* 2022, **29**(2)**:** 202-214.e207.

Figure S1

A

Antibodies used in the study

Protein	Clone	Company	Catalog No.
BCL2	SP66	Roche Diagnostics, Rotkreuz, Switzerland	06446329001
CD56	1B6	Leica Biosystems, Wetzlar, Germany	CD56-504-L-CE
Chromogranin A	DAK-A3	Agilent Technologies, Santa Clara, CA, USA	M086901-2
CK5	SP27	Roche Diagnostics	07292821001
MYC	Y69	Roche Diagnostics	06504612001
DLL3	SP347	Roche Diagnostics	08416931001
INSM1	A-8	Santa Cruz, Dallas, TX, USA	sc-271408
Ki-67	MIB1	Agilent technologies	M7240
KIT	Polyclonal	Agilent Technologies	A4502
p40	BC28	Roche Diagnostics	07394420001
POU2F3	Polyclonal	Sigma-Aldrich, St. Louis, MO, USA	HPA019652
Synaptophysin	MRQ-40	Roche Diagnostics	06433324001
TTF-1	8G7G3/1	Agilent Technologies	IR05661-2

B

Immunohistochemistry for POU2F3 in squamous cell carcinoma (SQCC) (our cohort)

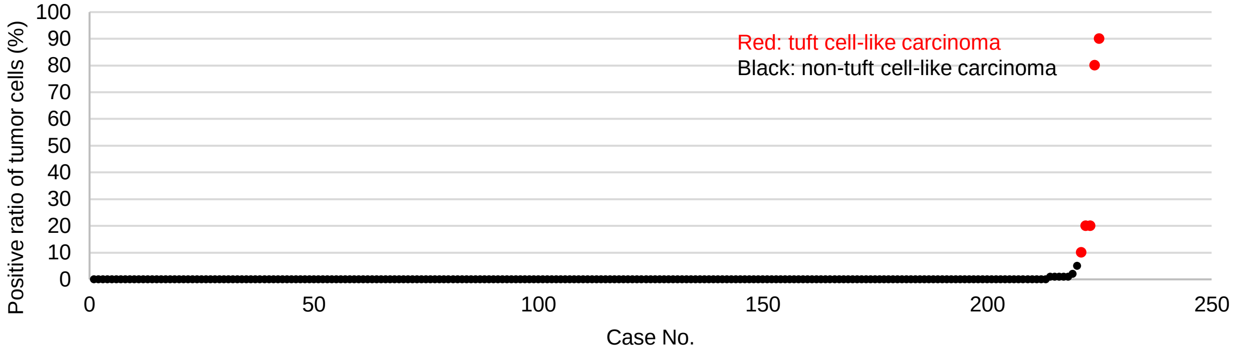

C

Primers used in the study

Gene	Forward (5' to 3')	Reverse (5' to 3')
Beta-2 microglobulin	actctctctttctggcctgg	gacaagtctgaatgctccact
BCL2	atcgccctgtggatgactgagt	gccaggagaaatcaaacagaggc
POU2F3	gctggagaagtttgccaagacc	gtgagatggtggtctggctgaa
PARP1	ccaagccagttcaggacctcat	ggatctgccttttgctcagcttc
PARP16	gcatttcatggtagccgcctag	caagtcactggtgaggtaggtc

Figure S2

Our data

A. Prognoses of patients with neuroendocrine carcinoma (SCLC + LCNEC) in cohort-J and cohort-G

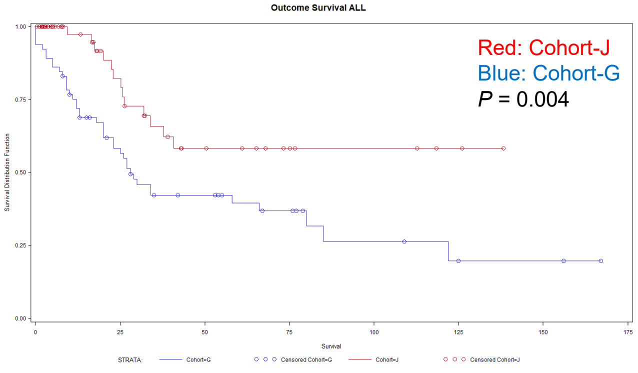

B. Factors influencing patient survival with *P* values

	Cohort-J	Cohort-G	Comments
SCLC vs. LCNEC	0,5032	0,1037	
Tuft cell-like vs. Non-tuft cell-like	0,0070	0,0464	Cohort-J: Worse in tuft<br>Cohort-G: Worse in non-tuft
Age (older vs. younger than mean [65.6 yo])	0,0503	0,9978	
Gender	0,3408	0,2443	
Size (larger vs. smaller than mean [22.6 mm])	0,1778	0,9304	
Ly (presence vs. absence)	0,1090	0,1561	
V (presence vs. absence)	0,0232	0,4592	Cohort-J: Worse in presence

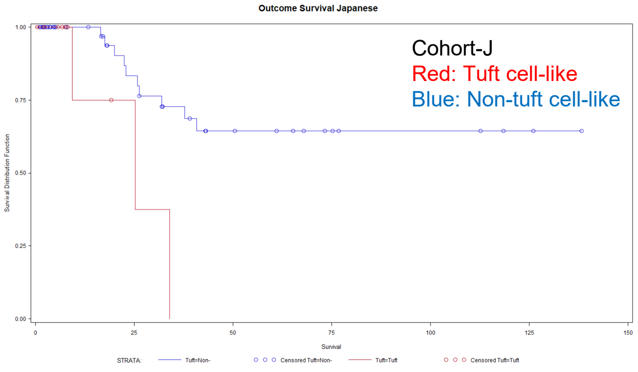

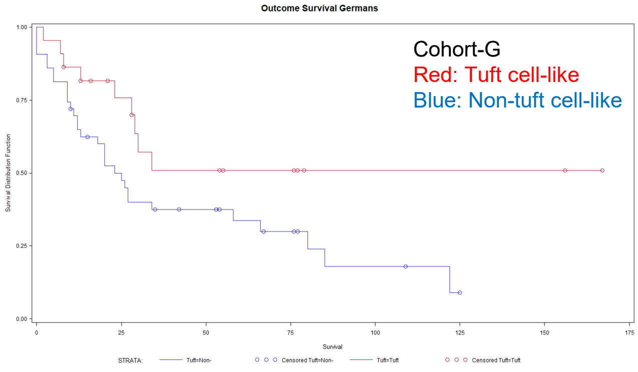

C. Multiple regression models

	Japanese	Germans	Comments
SCLC vs. LCNEC	0,0940		
Tuft cell-like vs. Non-tuft cell-like	0,3545	0,0464	
Age (older vs. younger than mean [65.6 yo])	0,0190		
V (presence vs. absence)	0,0401		
(SCLC vs. LSCNE) x (Tuft cell-like vs. Non-tuft cell-like)	0,0385		Tuft cell-like: w orse in LCNEC<br>Non-tuft cell-like: w orse in SCLC

Figure S3

Immunohistochemistry of tuft cell-like carcinomas (each number: positive ratio [%])

No.	Subtypes	POU2F3	CK5	CD56	BCL2	KIT	MYC	Ki67	CHGA	SYP	TTF1	INSM1	DLL3	p40
1	SCLC	20	0	80	20	10	10	50	0	0	20	1	0	0
2	SCLC	90	5	30	100	80	20	80	0	50	0	30	0	5
3	SCLC	80	40	0	100	80	0	50	0	0	0	NA	NA	0
4	SCLC	70	80	80	90	50	60	90	0	0	0	0	NA	5
5	SCLC	70	50	20	100	100	20	80	0	0	0	NA	NA	1
6	SCLC	70	20	70	100	80	10	80	0	5	1	70	NA	5
7	LCNEC	40	30	70	100	20	60	70	0	0	NA	NA	NA	NA
8	LCNEC	40	1	70	90	10	1	60	0	0	NA	NA	NA	NA
9	LCNEC	20	90	0	100	20	0	NA	30	40	NA	NA	NA	NA
10	LCNEC	90	1	20	100	10	20	90	0	1	0	NA	NA	0
11	LCNEC	10	20	10	40	10	10	80	0	5	0	NA	NA	0
12	LCNEC	60	10	50	90	20	30	70	0	30	0	NA	NA	NA
13	LCNEC	80	20	20	90	30	20	70	0	0	0	0	NA	2
14	SQCC	20	40	2	100	2	0	30	0	1	0	NA	NA	90
15	SQCC	80	100	90	90	40	10	20	20	50	1	NA	NA	100
16	SQCC	10	100	0	10	70	0	20	0	0	0	NA	NA	90
17	SQCC	20	100	10	40	20	0	30	0	0	0	NA	NA	70
18	SQCC	90	80	10	100	90	10	20	0	10	0	NA	NA	80

No.4: Combined SCLC (SCLC + spindle cell carcinoma)

No.6: Combined SCLC (SCLC + SQCC)

Figure S4

Our cohort (cohort-J): tuft cell-like small cell lung cancer (SCLC)

A

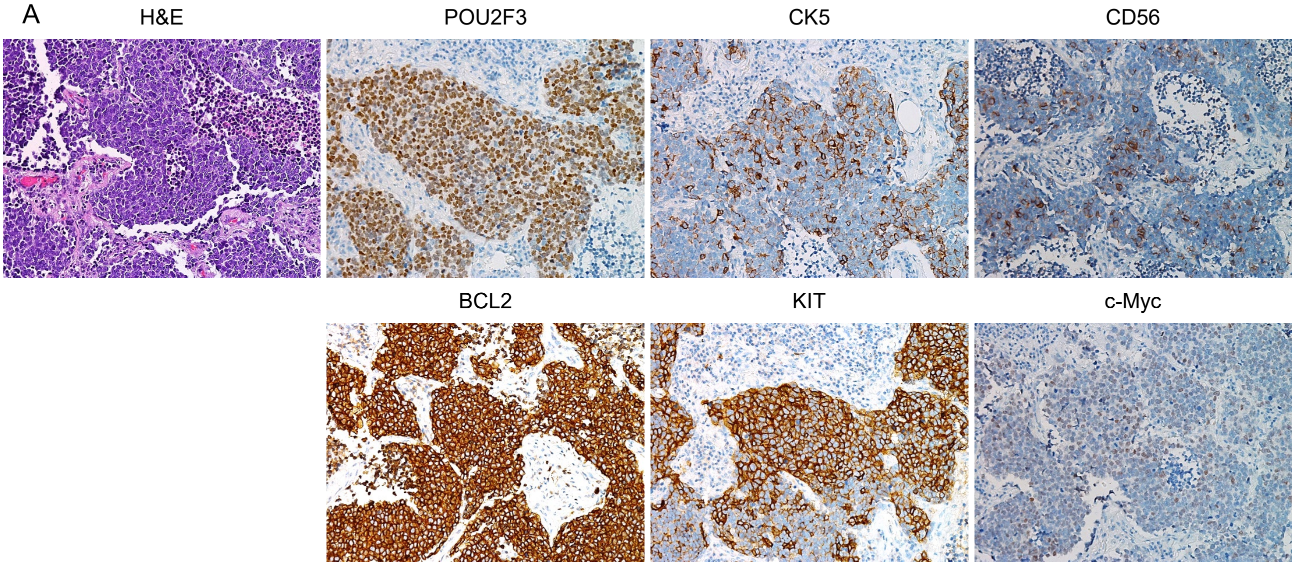

B

SCLC (tuft cell-like, 6; non-, 30)

	Tuft	Non-	*P*
Age	73.0	68.9	0.30
Gender			0.61
Male	4	23	
Female	2	7	
Smoking			0.21
current	1	16	
ex	4	11	
never	0	2	
Pack-year	60.8	47.7	0.32
Histology			0.23
Pure	4	26	
Combined	2	4	
Size (mm)	19.0	25.0	0.32
Pl			0.33
0	5	15	
1-3	1	9	
Ly			0.18
ly0	6	19	
ly1	0	6	
V			0.85
v0	3	11	
v1	3	13	
pT			0.24
1	6	19	
2	0	8	
3	0	2	
4	0	0	
pN			0.16
0	5	9	
1-3	0	21	
pStage (UICC 8th)			0.22
I	5	18	
II	0	9	
III	0	3	
IV	0	0	

C

Patients' prognosis with SCLC

Red: tuft cell-like, blue: non-tuft cell-like

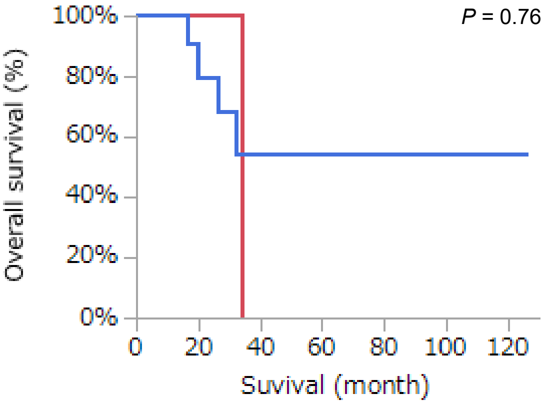

D

Patients' prognosis with tuft cell-like cancers

Red: tuft cell-like SCLC, blue: tuft cell-like LCNEC

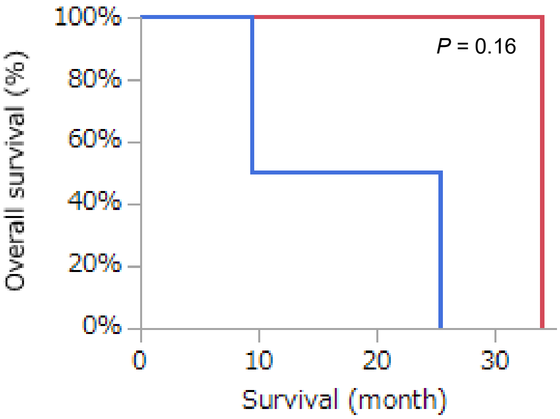

Figure S5

Our cohort (cohort-J): tuft cell-like squamous cell carcinoma (SQCC)

A

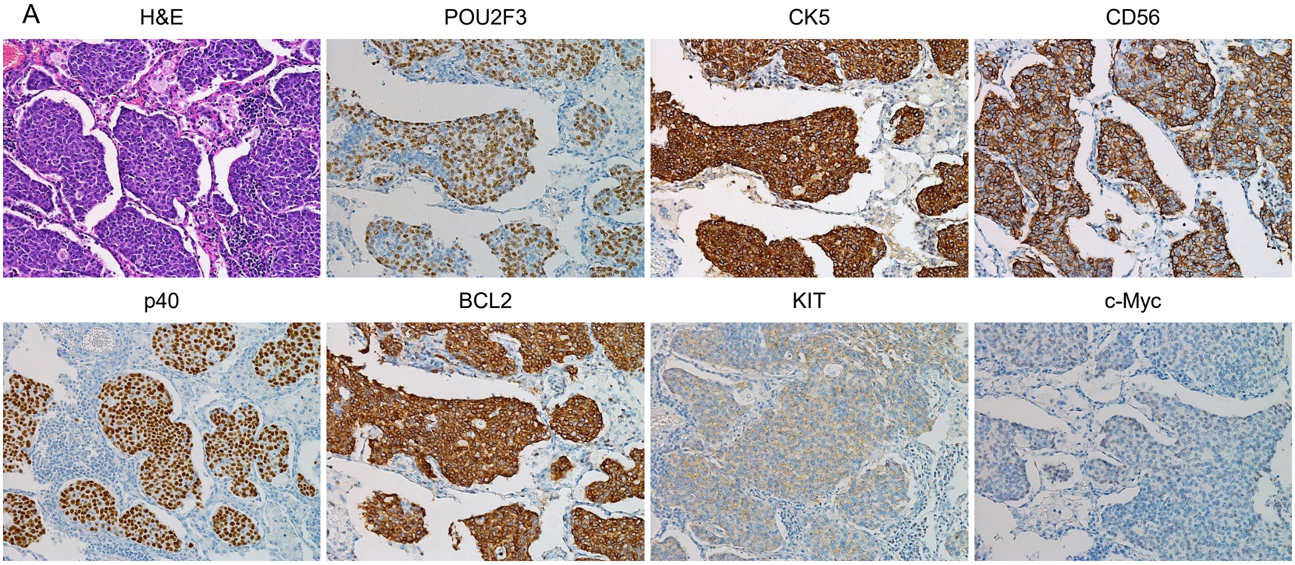

B

SQCC (tuft cell-like, 5; non-, 220)

	Tuft	Non-	*P*
Age	68.0	71.4	0.31
Gender			0.66
Male	4	191	
Female	1	29	
Smoking			0.11
current	1	112	
ex	3	100	
never	1	8	
Pack-year	67.9	63.5	0.79
Differentiation			0.14
Wel-mod	2	155	
Poor	3	65	
Size (mm)	28	33	0.48
Pl			0.36
p0	4	131	
p1-3	1	89	
V			0.31
v0	4	126	
v1	1	94	
Ly			0.051
ly0	3	195	
ly1	2	25	
pT			0.58
1	3	90	
2	2	68	
3	0	38	
4	0	24	
pN			0.56
0	3	158	
1-3	2	62	
pStage (UICC 8th)			0.65
I	3	109	
II	2	58	
III	0	52	
IV	0	1	

C

Patients' prognosis with SQCC

Red: tuft cell-like, blue: non-tuft cell-like

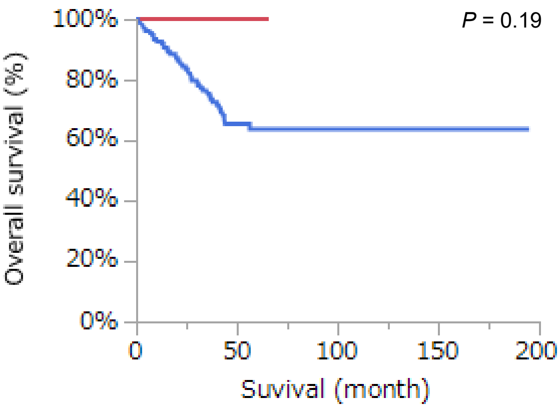

Figure S6

Our cohort (cohort-G)

A. Clinicopathological features of tuft cell-like lung cancers

**SCLC (tuft cell-like, 18; non-, 29)**	Tuft	Non-	*P*	**LCNEC (tuft cell-like, 6; non-, 17)**	Tuft	Non-	*P*
Age	64.8	62.8	0.50	Age	63.8	63.3	0.90
Gender			0.89	Gender			0.11
Male	13	17		Male	2	12	
Female	5	12		Female	4	5	
Size (mm)	14.5	9.0	0.02	Size (mm)	10.0	11.0	1.00
Ly			0.04	Ly			1.00
ly0	6	11		ly0	4	8	
ly1	5	1		ly1	1	2	
V			0.69	V			0.46
v0	10	12		v0	5	9	
v1	1	2		v1	0	1	
pT			0.93	pT			0.26
1	13	21		1	3	5	
2	3	4		2	2	3	
3	1	1		3	0	7	
4	0	0		4	1	1	
pN			0.97	pN			0.48
0	8	13		0	3	10	
1-3	6	10		1-3	3	5	

B.
Patients' prognosis with SCLC

Red: tuft cell-like
blue: non-tuft cell-like

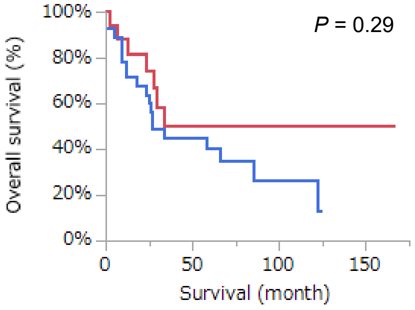

C.
Patients' prognosis with LCNEC

Red: tuft cell-like
blue: non-tuft cell-like

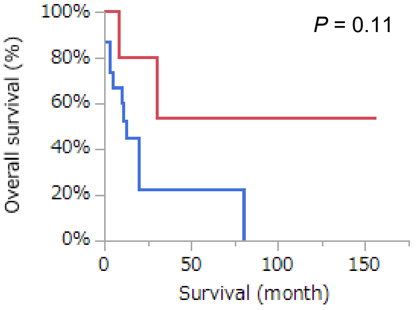

D.
Patients' prognosis with tuft cell-like cancers

Red: tuft cell-like SCLC
blue: tuft cell-like LCNEC

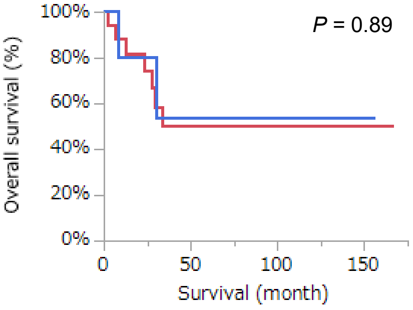

E. Immunohistochemisry (IHC) for small cell lung cancer (SCLC) (Tuft cell-like, 18; Non-tuft cell-like, 29)

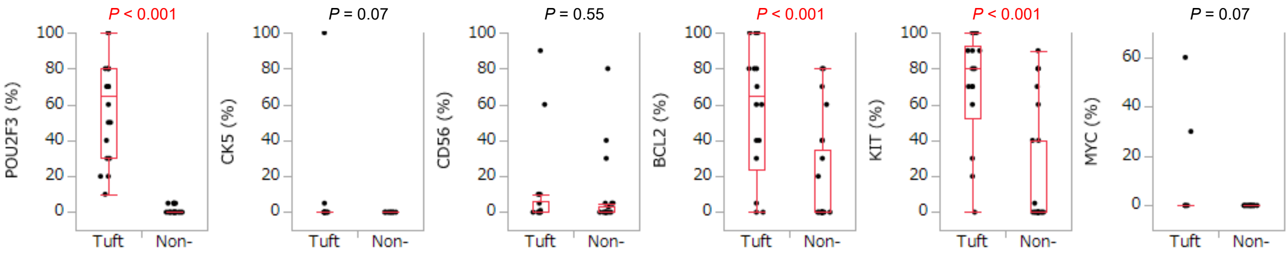

F. IHC for large cell neuroendocrine carcinoma (LCNEC) (Tuft cell-like, 6; Non-tuft cell-like, 17)

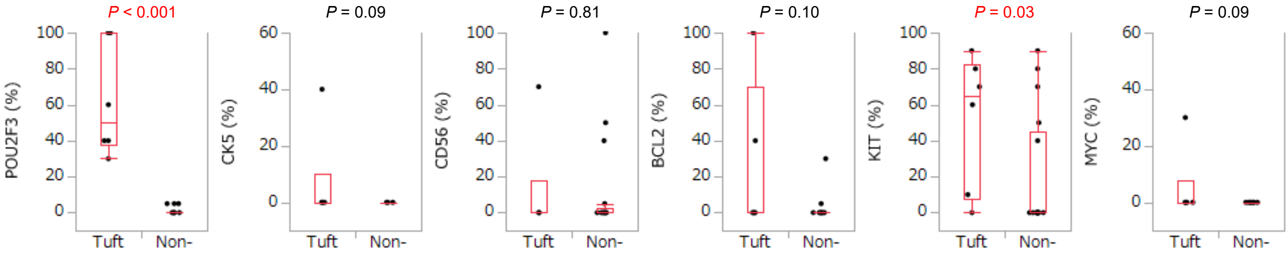

Figure S7

A

Tuft cell-like lung cancers diagnosed as adenocarcinoma and SQCC (TCGA, Nature 2014 and 2012)

Subtype	SAMPLE_ID	POU2F3	GFI1B	TRPM5	SOX9	CHAT	ASCL2	AVIL
Adenocarcinoma	TCGA-50-5931-01	13.1661	47.0204	0.6259	2.0005	-0.2103	11.3277	0.5524
SQCC	TCGA-39-5035-01	22.7068	2.269	6.6036	-0.6169	0.0408	6.3035	12.3564
SQCC	TCGA-43-6143-01	50.9897	15.1403	6.4503	1.6649	0.0999	5.5092	0.0786
SQCC	TCGA-66-2757-01	11.5504	10.3543	7.5274	5.6345	0.576	14.7821	4.2777

-3 3

B. mRNA expression of SCLC (George et al., 2015)

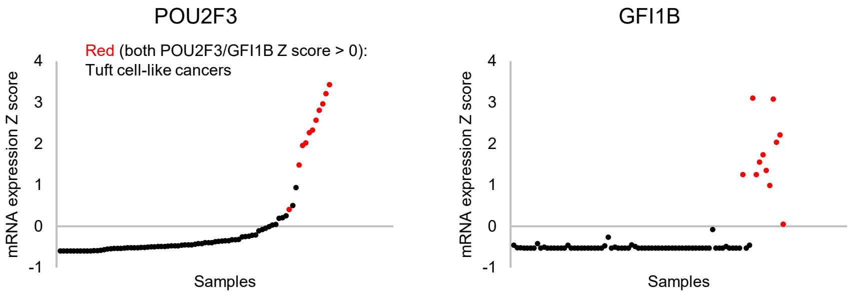

mRNA expression of tuft cell markers in tuft cell-like SCLCs (George et al., 2015)

SAMPLE_ID	POU2F3	GFI1B	TRPM5	SOX9	CHAT	ASCL2	AVIL
sclc_ucologne_2015_S00829	2.0182	1.557	0.6275	0.2221	-0.1843	1.5578	2.29
sclc_ucologne_2015_S00837	1.4841	3.106	1.9029	2.1463	-0.2842	1.7505	2.3554
sclc_ucologne_2015_S01542	1.9614	1.2572	1.1057	2.0035	-0.2842	2.9318	0.0839
sclc_ucologne_2015_S01556	3.2171	2.2119	3.5118	2.6105	0.0388	1.6789	3.63
sclc_ucologne_2015_S02209	0.4134	1.2536	2.1884	1.2021	-0.2842	1.9529	-0.8143
sclc_ucologne_2015_S02255	3.4291	0.0557	4.1912	0.0506	-0.181	2.7977	4.1249
sclc_ucologne_2015_S02256	2.8122	3.0844	-0.2126	3.0715	-0.1868	1.3164	2.1566
sclc_ucologne_2015_S02286	2.5685	0.9878	3.1328	1.9022	-0.2083	2.1706	0.6269
sclc_ucologne_2015_S02288	2.3278	1.3522	0.4975	1.5298	-0.2842	1.449	-0.2579
sclc_ucologne_2015_S02296	2.9668	2.0346	3.112	0.9184	-0.2638	1.0343	3.0052
sclc_ucologne_2015_S02375	2.2647	1.7363	2.1239	1.6258	-0.283	2.5162	1.3635

-3 3

C. mRNA expression of LCNEC (George et al., 2018)

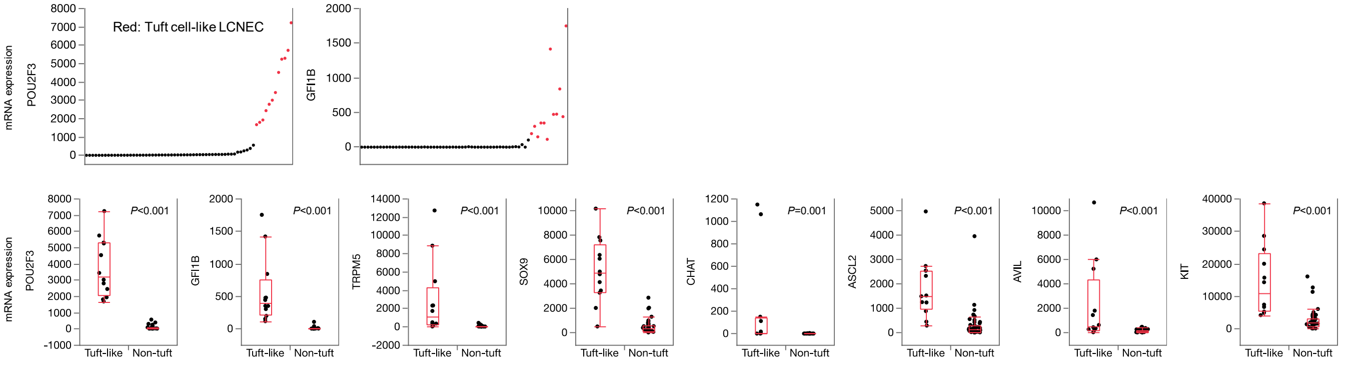

Figure S8

A. mRNA expression in SQCC (RNA seq Z score) (TCGA, Nature 2012)

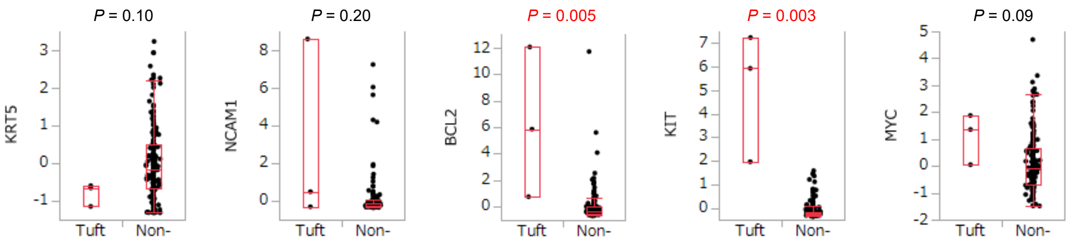

B. Patients' prognosis with tuft cell-like NECs (George et al., 2015, 2018)

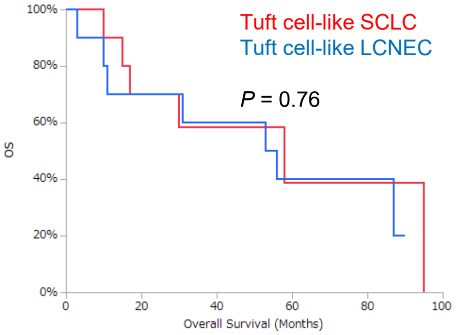

C-D. Differentially expressed genes between tuft cell-like SCLC and LCNEC (George et al., 2015, 2018)

C

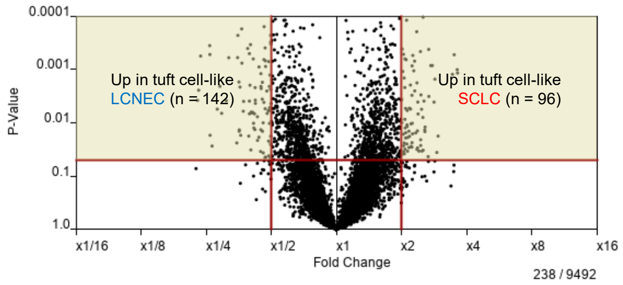

D

Upregulated in tuft cell-like (tcl-)SCLC compared to tcl-LCNEC

Rank	Category	P-value
1	response to virus	4.E-07
2	type i interferon signaling pathw ay	6.E-07
3	cellular response to tumor necrosis factor	9.E-07
4	cellular response to interleukin-1	1.E-06
5	response to interferon-gamma	2.E-06
6	anterior/posterior pattern specification	5.E-06
7	positive regulation of protein kinase b signaling	9.E-06
8	negative regulation of viral genome replication	3.E-05
9	monocyte chemotaxis	4.E-05
10	positive regulation of chemotaxis	6.E-05

Upregulated in tuft cell-like (tcl-)LCNEC compared to tcl-SCLC

Rank	Category	P-value
1	neuron fate specification	3.E-06
2	central nervous system development	4.E-05
3	glial cell differentiation	1.E-04
4	perikaryon	2.E-04
5	neuron differentiation	3.E-04
6	presynapse	3.E-04
7	endochondral ossification	8.E-04
8	cerebral cortex development	8.E-04
9	embryonic limb morphogenesis	2.E-03
10	transport vesicle membrane	2.E-03

E. mRNA expression of ionocyte-related genes in tuft cell-like cancers (George et al., 2015, 2018, TCGA Nature 2012)

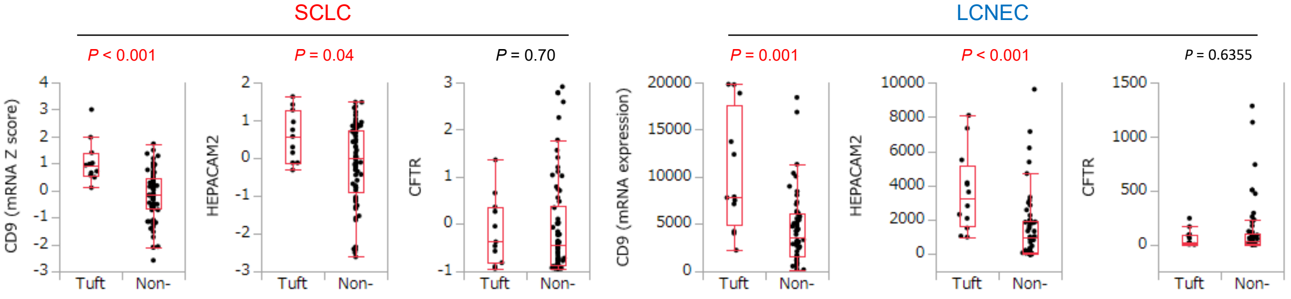

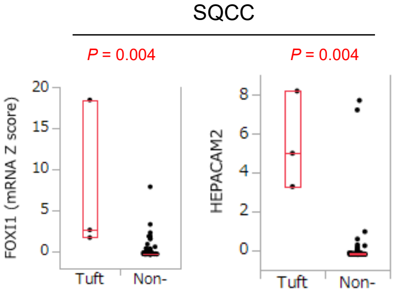

Figure S9

Our data

A

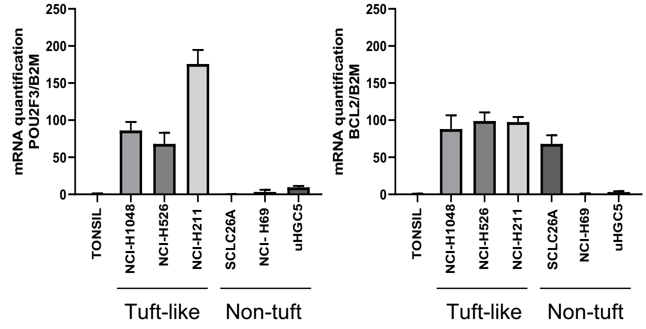

B

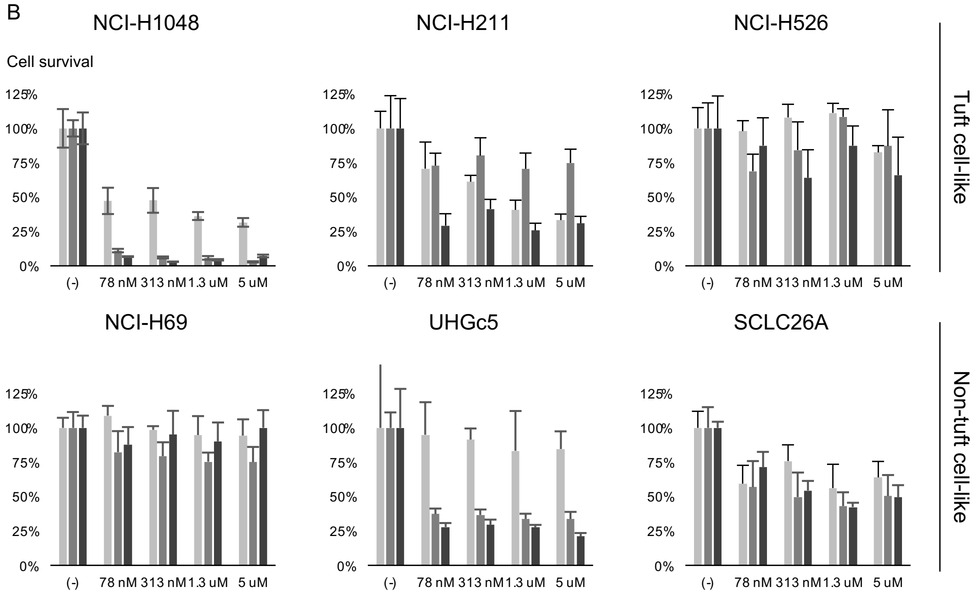

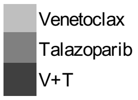

IC50 (uM)				
Cell lines	V	T	Mix	CI
NCI-H1048	0.07	0.0001	2.64.E+08	1.32E+12
NCI-H211	0.73	0.0003	NC	NC
NCI-H526	6.57	0.04	0.0003	0.004
NCI-H69	24.8	15.5	10.8	0.57
UHGc5	5.38	NC	NC	NC
SCLC2617	NC	0.06	1.35	NC

CI: combination index

NC: Could not be calculated

C

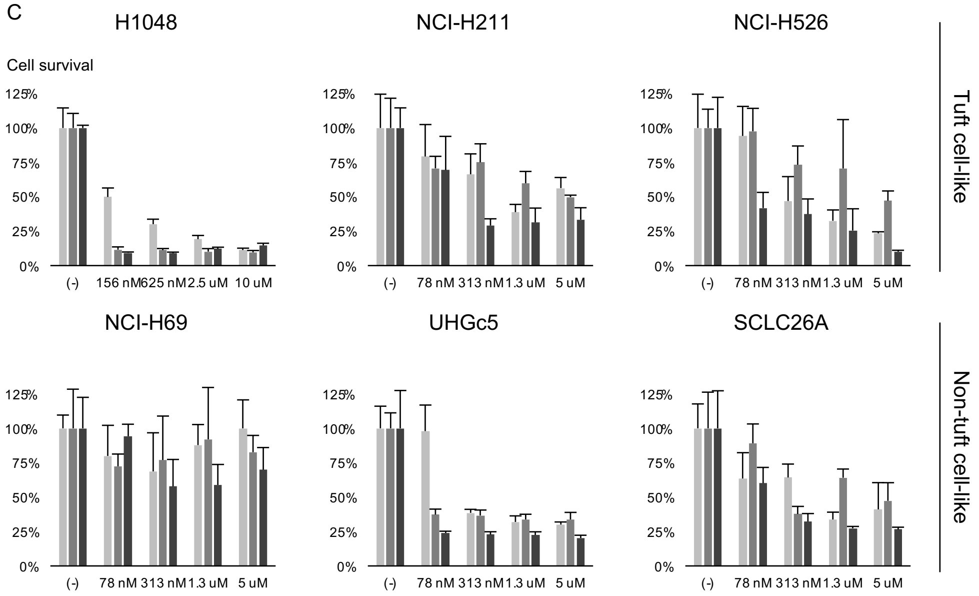

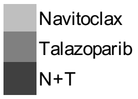

IC50 (uM)				
Cell lines	N	T	Mix	CI
NCI-H1048	0.12	NC	544	NC
NCI-H211	0.08	0.14	0.03	0.25
NCI-H526	0.51	4.25	0.05	0.05
NCI-H69	18.3	0.004	0.01	1.68
UHGc5	0.48	NC	NC	NC
SCLC2617	0.68	NC	0.12	NC

CI: combination index

NC: Could not be calculated

Figure S10

Our data

A

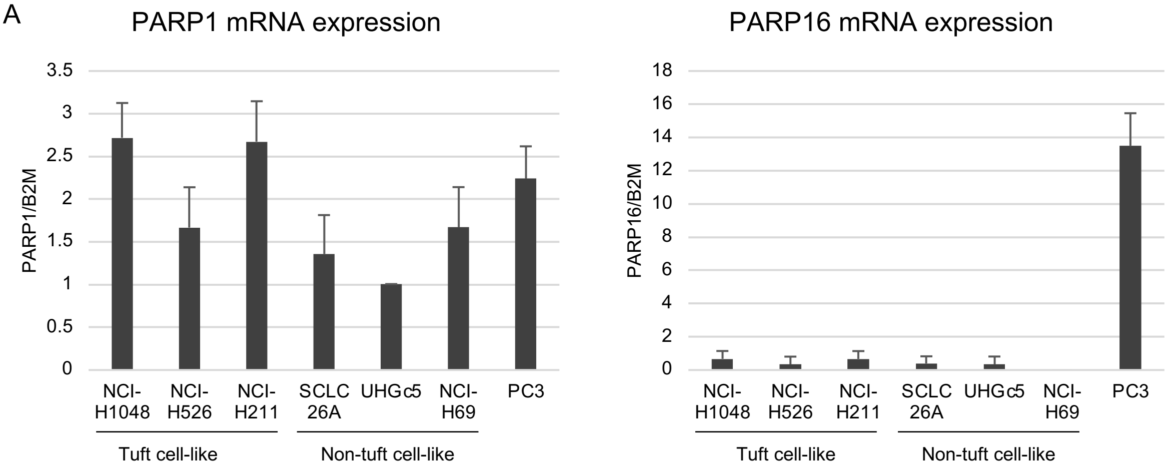

B

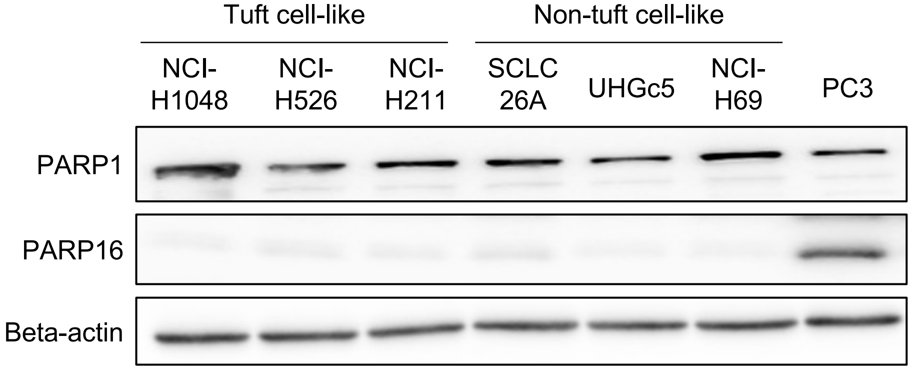

Supplement: Supplementary file 1 — Supplementary information [file 41419_2022_5428_MOESM1_ESM.pdf]
