# Supplementary material for: Pulmonary cancers across different histotypes share hybrid tuft cell/ionocyte-like molecular features and potentially druggable vulnerabilities
Source: Cell Death Dis. 2022 Nov 19;13(11):979. doi: 10.1038/s41419-022-05428-x (PMC9675833; doi:10.1038/s41419-022-05428-x)

Figure 5C. POU2F3

S597: POU2F3-expressing small cell lung cancer cell line (this cell line was not used in our study)

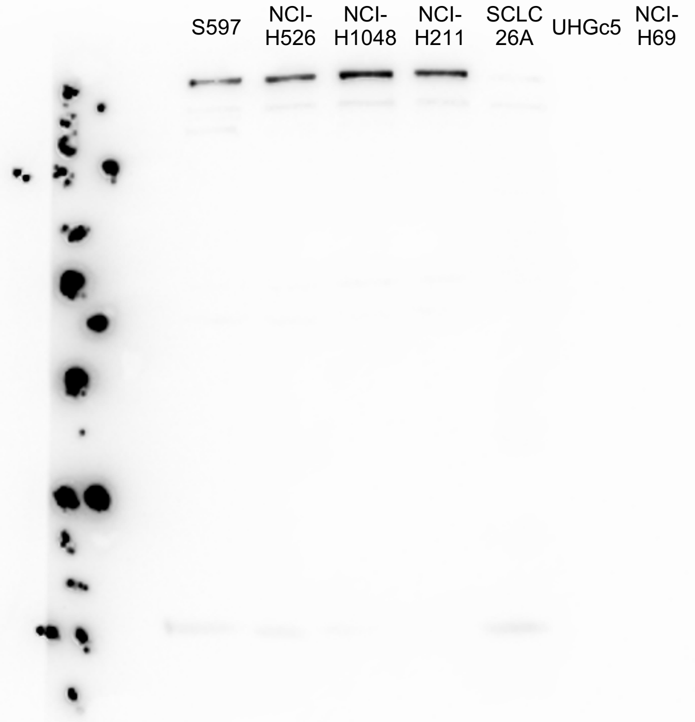

Note: We never see the marker in the exposition; When we switch the normal light and fix the membrane, we do see the Marker and we label the positions of the 3 mean bands on it in this case (75, 45 and 25) on the membrane, we then close the door and expose our membrane. We checked them several times to recognize the right bands.

Figure 5C. BCL2

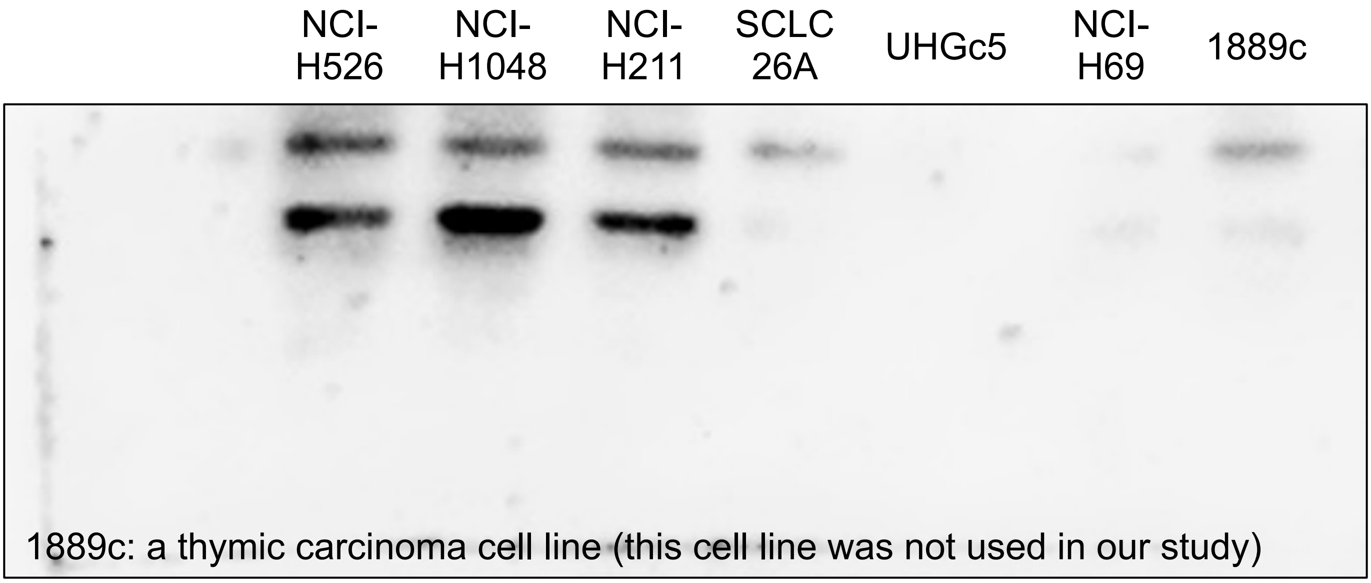

Figure 5C. B-actin

1889c, MP57:
thymic carcinoma cell lines (these cell lines were not used in our study)

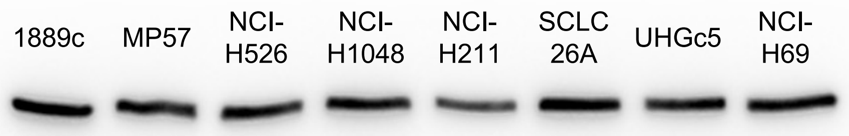

Figure S10B. PARP1

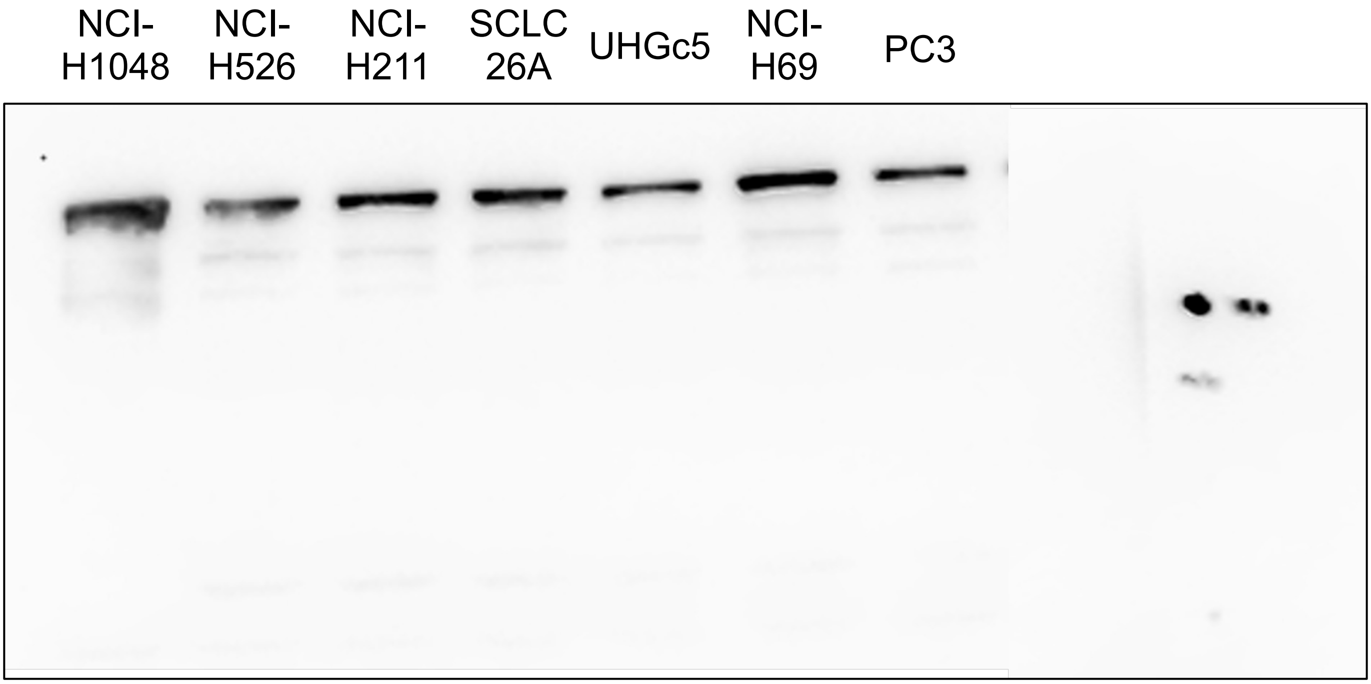

Figure S10B. PARP16

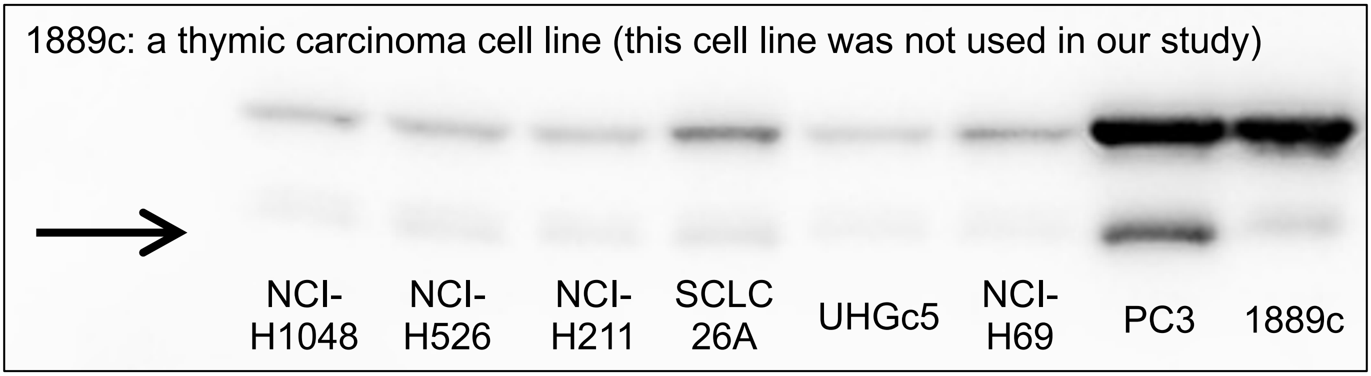

Figure S10B. Beta-actin

1889c: a thymic carcinoma cell line (this cell line was not used in our study)

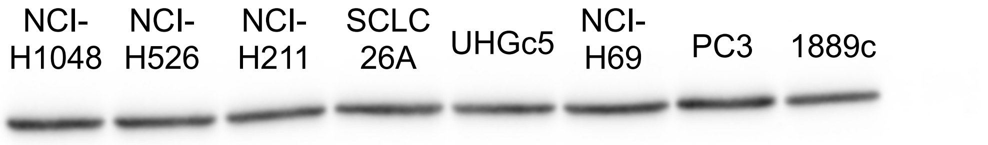

Supplement: Supplementary file 2 — Raw data of WB of Fig. 5 and S10 [file 41419_2022_5428_MOESM2_ESM.pdf]
